# The arcuate fasciculus: Combining structure and function into surgical considerations

**DOI:** 10.1002/brb3.3107

**Published:** 2023-06-06

**Authors:** Laura Vavassori, Martina Venturini, Luca Zigiotto, Luciano Annicchiarico, Francesco Corsini, Paolo Avesani, Laurent Petit, Alessandro De Benedictis, Silvio Sarubbo

**Affiliations:** ^1^ Department of Neurosurgery Azienda Provinciale per i Servizi Sanitari (APSS), “S. Chiara” Hospital Trento Provincia Autonoma di Trento Italy; ^2^ Center for Mind and Brain Sciences (CIMeC) University of Trento Trento Provincia Autonoma di Trento Italy; ^3^ Neuroinfrmatics Laboratory (NiLab) Bruno Kessler Foundation Povo Provincia Autonoma di Trento Italy; ^4^ Groupe d'Imagerie Neurofonctionnelle, Institut des Maladies Neurodégénératives (GIN‐IMN), UMR5293, CNRS, CEA University of Bordeaux Bordeaux France; ^5^ Neurosurgery Unit, Department of Neurosciences Bambino Gesù Children's Hospital IRCCS Rome Italy

**Keywords:** arcuate fasciculus, function, glioma resection, structure, surgical approaches, white matter

## Abstract

**Background:**

Two Centuries from today, Karl Friedrich Burdach attributed the nomenclature *“arcuate fasciculus”* to a white matter (WM) pathway connecting the frontal to the temporal cortices by arching around the Sylvian fissure. Although this label remained essentially unvaried, the concepts related to it and the characterization of the structural properties of this bundle evolved along with the methodological progress of the past years. Concurrently, the functional relevance of the arcuate fasciculus (AF) classically restricted to the linguistic domain has extended to further cognitive abilities. These features make it a relevant structure to consider in a large variety of neurosurgical procedures.

**Objective:**

Herein, we build on our previous review uncovering the connectivity provided by the Superior Longitudinal System, including the AF, and provide a handy representation of the structural organization of the AF by considering the frequency of defined reports in the literature. By adopting the same approach, we implement an account of which functions are mediated by this WM bundle. We highlight how this information can be transferred to the neurosurgical field by presenting four surgical cases of glioma resection requiring the evaluation of the relationship between the AF and the nearby structures, and the safest approaches to adopt.

**Conclusions:**

Our cumulative overview reports the most common wiring patterns and functional implications to be expected when approaching the study of the AF, while still considering seldom descriptions as an account of interindividual variability. Given its extension and the variety of cortical territories it reaches, the AF is a pivotal structure for different cognitive functions, and thorough understanding of its structural wiring and the functions it mediates is necessary for preserving the patient's cognitive abilities during glioma resection.

## Graphical abstract

The descriptions attributed to the term “Arcuate Fasciculus” considerably evolved since its first mention in Burdach's work; The extension of the Arcuate Fasciculus accounts for its large functional potentiality and underpins the necessity of its preservation during surgical procedures; A thorough understanding of its structural wiring and functional implications is of foremost importance for surgical planning.

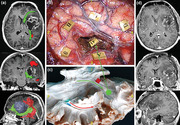

## 1 INTRODUCTION

Most of the vocabulary that we use to refer to the components of the nervous system and the related mechanisms comes from early anatomical studies performed between the 18th and the 19th Century. Karl Friedrich Burdach (1776–1847) is among the main contributors to our established neuroanatomical lexicon (Swanson, 2015). Considering his studies on the cerebral white matter (WM), his legacy entails the identification and labeling of the main association bundles of the human brain (Burdach, 1826). The second volume of *“Vom Baue und Leben des Gehirns*,*”* published in 1822, contains the first mention of the name *“arcuate fasciculus”* (AF)—originally *“Bogenbündel”* (i.e., arcuate fascicle) (Burdach, 1822). Owing to this description, Burdach's name has historically been associated to this WM bundle, to the extent that the Dejerines referred to it as *“faisceau arqué de Burdach”* (i.e., arcuate fascicle of Burdach (Dejerine & Dejerine‐Klumpke, 1895, 1901).

Two hundred years went by, and we still use Burdach's denomination. What changed is the definition of this WM structure, including the attributed partonomies, subdivisions, and the respective nomenclatures (Mandonnet et al., 2018; Porto de Oliveira et al., 2021; Vavassori et al., 2021). Indeed, the introduction and fast evolution of neuroimaging techniques (Leemans, 2019), the revisitation of early neuroanatomical discoveries (De Benedictis et al., 2014; Hope et al., 2016; Sarubbo et al., 2016), and the renovation of Klingler's microdissection approach (Agrawal et al., 2011; Klingler, 1935; Martino et al., 2011) led to the production of increasingly more detailed descriptions of the human WM pathways. In particular, the dissemination of diffusion magnetic resonance imaging‐based tractography (Basser et al., 2000) enabled the first characterization of the arcuate fascicle in vivo (Catani et al., 2002), leading off a series of innovative studies aimed to achieve the most accurate and comprehensive characterization of the wiring and extension of this bundle. Concurrently with these efforts, the ensuing functional relevance of the AF has become steadily more evident: damages of this bundle, both in terms of altered microstructural features or disconnection, have been implicated in a broad spectrum of syndromes, spanning from psychiatric symptoms (Jiang et al., 2017; Psomiades et al., 2016) to neurological diseases (Nakajima et al., 2018). Moreover, its volume in the right hemisphere has been indicated as a reliable predictor of aphasia recovery after stroke (Forkel et al., 2014). The involvement of the AF in such a variety of syndromes, and therefore of associated cognitive processes, relates to its large volume and long‐range extension. Indeed, the AF transverses three lobes (i.e., the frontal, parietal, and temporal cortices) and resides in close relationship with the insula, with longitudinal associative fascicles laterally (Mandonnet et al., 2018) and projection fibers on the medial side. These characteristics make it a fundamental mediator of different cognitive abilities and explain why it must inevitably be considered in many diverse surgical procedures.

The continuous methodological evolutions and the interest of the scientific community in investigating the properties of this specific WM bundle surely contributed to the achievement of a more complete depiction of the AF, but they also led to the production of an intricate and non‐univocal picture (Becker et al., 2022; Mandonnet et al., 2018; Porto de Oliveira et al., 2021; Vavassori et al., 2021). Therefore, an overview of the complex literature about this bundle is essential to highlight its multiple structural and functional facets that need to be considered when approaching the study of this bundle, especially in the surgical practice. By analyzing the controversies regarding the Superior Longitudinal System's subdivisions and the respective nomenclatures, our recent literature review (Vavassori et al., 2021) already exposed the current knowledge about the structural wiring of the AF intended as a whole ensemble of fibers connecting the frontal and the temporal lobes of the same hemisphere by passing above the insula (Mandonnet et al., 2018). The present work builds on this previous examining the architecture of frontotemporal dorsal associational connections independently from their partonomy or labeling and integrates it by addressing the functional roles attributed to the AF. We adopt a cumulative approach to reshape the structural definition of the bundle and to present the cognitive processes it mediates. This new perspective provides an easy‐to‐access general characterization of the AF, and by supplying supplementary information about the frequency of certain descriptions accounts for the most common as well as for the rarest patterns of connectivity/functional responses that should be considered in the surgical framework when dealing with this fasciculus. Indeed, emerging knowledge in the field of connectomics has become relevant for surgical approaches to the resection of tumors, highlighting the importance of the AF preservation while balancing between the extension of resection and the upkeep of the patient's quality of life (Bunevicius et al., 2014; Hervey‐Jumper & Berger, 2016; Sanai et al., 2011; Zarino et al., 2020; Zigiotto et al., 2020). With this aim, we append to our literature review a description of surgical approaches to tumor resection that require the careful consideration of this bundle extension, its relationships with the nearby anatomical structures and the related functional implications, and provide a collection of four different surgical cases to highlight how, in practice, extensive knowledge of the AF features can be applied in this field.

## 2 OLD AND NEW INSIGHTS ABOUT THE AF

*From its first accounts…*

**FIGURE 1:**
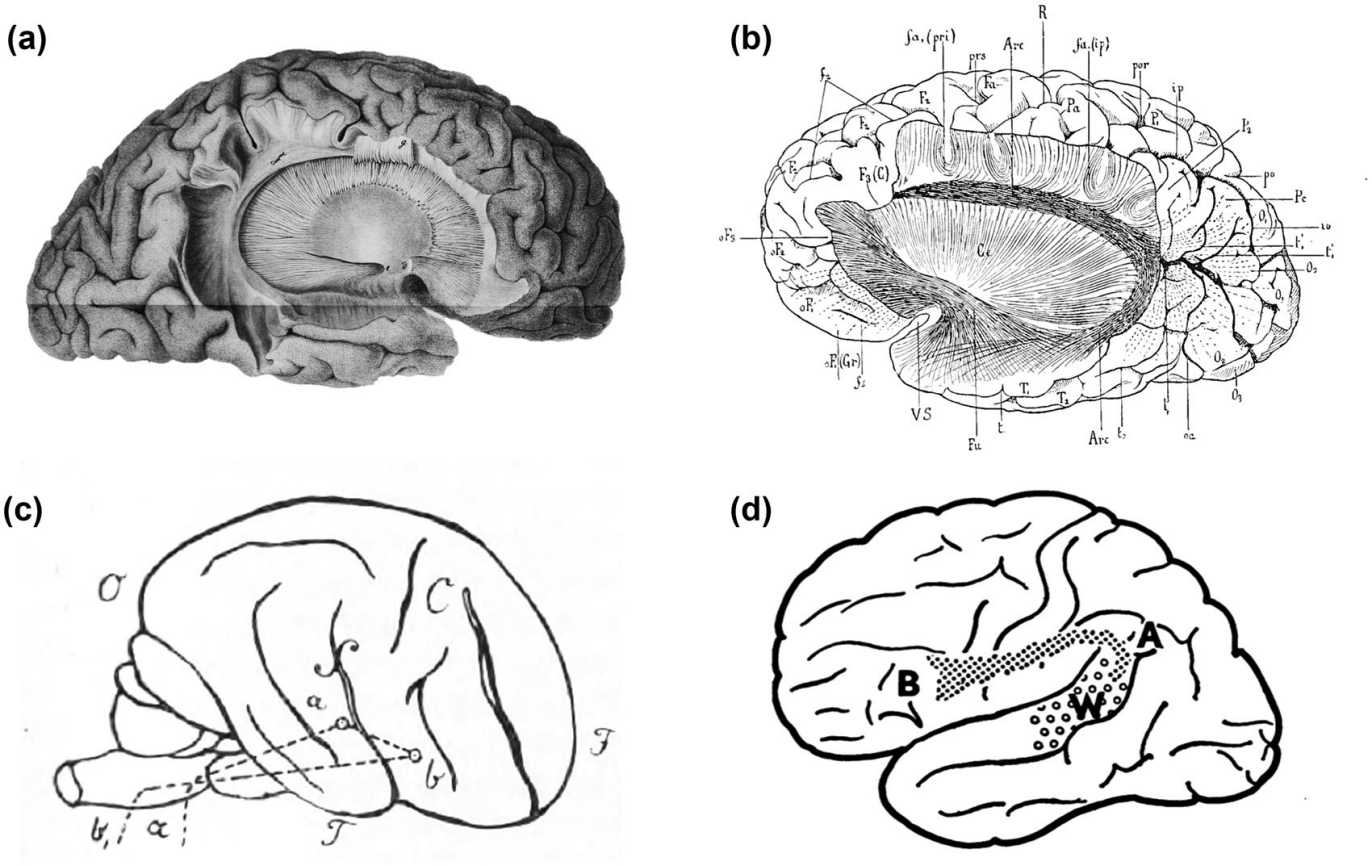
Historical drawings and diagrammatic representations of the arcuate fasciculus (AF) from the original works of (a) Reil (1812), (b) Dejerine and Dejerine‐Klumpke (1895), (c) Wernicke (1874), and (d) Geschwind (1970).

The classification of the association pathways of the human brain was fostered by the works of pioneering dissectionists who, starting from the early 19th Century up to the beginning of the 20th Century, carried out a systematic exploration of the WM structural organization (Burdach, 1826; Dejerine & Dejerine‐Klumpke, 1895, 1901; Mayo, 1823; Reil, 1809, 1812; Monakow, 1897). In this instance, different anatomists reported that an orderly ensemble of fibers analogous to the more medial cingulum bundle could be isolated on the lateral aspect of each cerebral hemisphere (Burdach, 1822; Dejerine & Dejerine‐Klumpke, 1895, 1901). This WM pathway was described and finely illustrated as a connection running between the temporal and the frontal lobes, passing through the parietal lobe, and arching around the posterior limit of the Sylvian fissure (Dejerine & Dejerine‐Klumpke, 1895; Reil, 1812) (Figure 1a,b). Its characteristic arched shape granted it the name *“arcuate fasciculus”* (Burdach, 1822). In this same period, based on the observation of clinical signs related to specific anatomical lesions (Kumar et al., 2011), Carl Wernicke proposed a putative model for the localization of linguistic abilities in the brain: following a previous description of the faculty of speech production being located in the third frontal convolution of the left hemisphere (i.e., *pars triangularis* (IFGtri) and *pars opercularis* (IFGop) of the inferior frontal gyrus (IFG)) (Broca, 1865), he advanced that a second area, later established to be represented by the superior temporal gyrus (STG), would support words’ sound memory, and therefore language comprehension (Wernicke, 1874). This characterization of the distribution of linguistic abilities led Wernicke himself to postulate that a specific syndrome characterized by spared comprehension with repetition deficits, and defined as conduction aphasia, could arise from a disconnection between these two cortical regions (Figure 1c). In the 1960s, Geschwind formalized that the anatomical substrate for the interaction between Wernicke and Broca's areas is represented by the AF (Geschwind, 1965) (Figure 1d).

*… to its current definition*

Nowadays, it would be reductive to define the AF as a connection running exclusively between the IFG and the STG and purely devoted to language comprehension. Especially throughout the last 20 years, the creation and progressive fine‐tuning of innovative techniques for the in vivo representation of the brain's WM and the testing of cognitive functions dramatically enriched the characterization of the AF.

**FIGURE 2:**
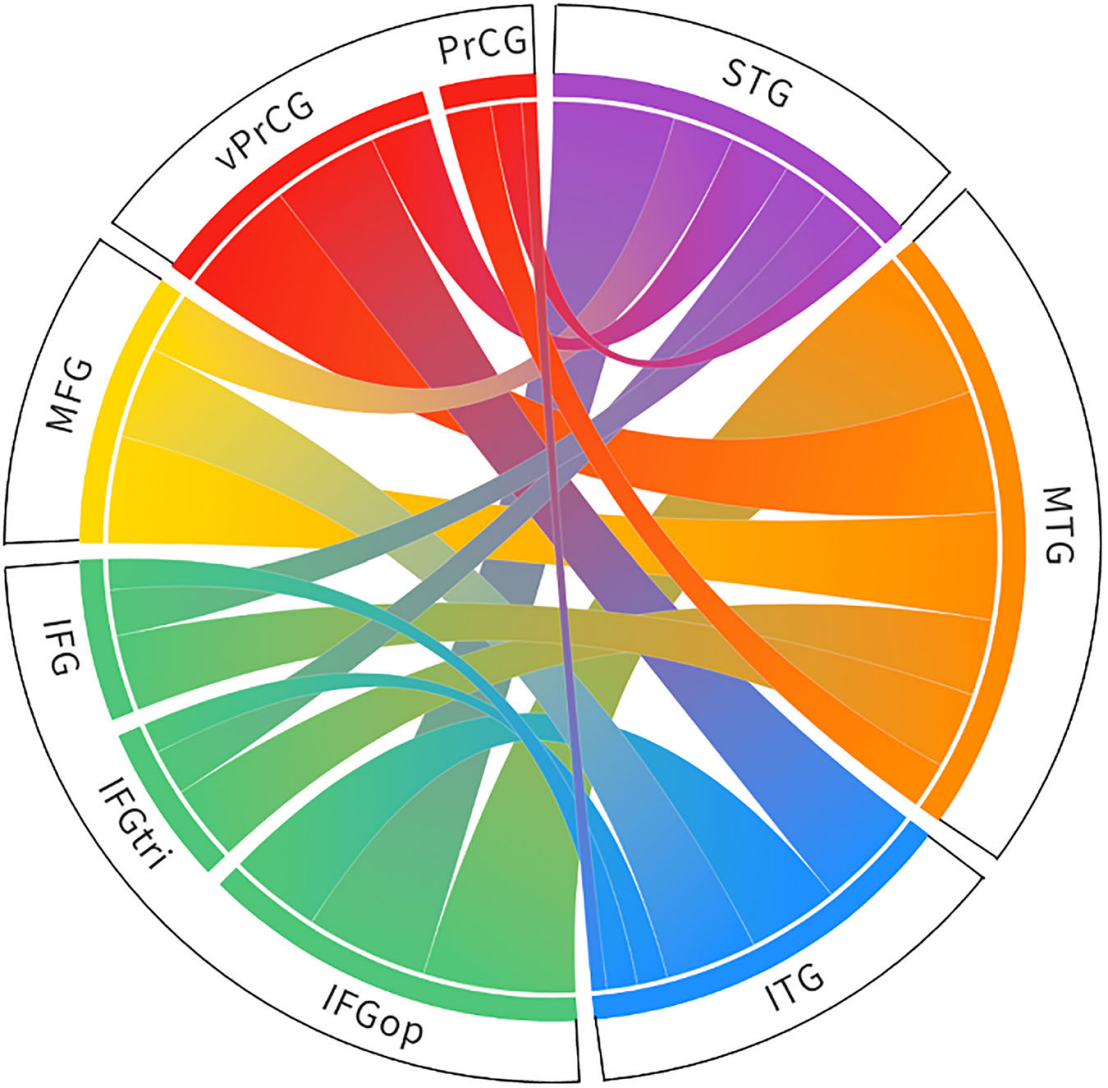
Representation of the frontotemporal connectivity provided by the arcuate fasciculus (AF) according to the literature reviewed in Vavassori et al. (2021). In line with the present cumulative perspective, the width of each link is proportional to the number of studies referring to the AF and mentioning a certain pattern of inter‐gyral connectivity. IFG, inferior frontal gyrus; IFGop, pars opercularis; IFGtri, pars triangularis; ITG, inferior temporal gyrus; MFG, middle frontal gyrus; MTG, middle temporal gyrus; PrCG, precentral gyrus; STG, superior temporal gyrus; vPrCG ventral precentral gyrus.

As mentioned above, an exhaustive account of the current definition of the AF structural wiring can be found in Vavassori et al. (2021) considering all the connections running between the frontal and temporal lobes, independently from the adopted nomenclature. Herein, we set the debate on the bundle's partitioning aside and build on these data by analyzing how frequently defined connections have been attributed to the AF description across studies. The present approach aims to highlight the most characterizing patterns of connectivity established by the AF (i.e., those that are more likely to be found when approaching the study of this bundle), while still representing those that have been reported only in few instances. This provides an account of the possible differences in the AF extension across individuals, an important variable that should be accounted for especially in the clinical setting (Forkel et al., 2021). Since very few studies explicitly analyze and report a specific pattern of connectivity between two precise cortical regions and the majority of them rather indicate the overall anterior and posterior terminations of the bundle, we assume all the areas listed in the same description to be mutually interconnected unless differently specified (Figure 2, see Table 1 for references).

**TABLE 1:** Patterns of the arcuate fasciculus (AF) structural connectivity as defined in the studies reviewed in Vavassori et al. (2021) and classified according to the present cumulative approach.

**Frontal termination**	**Temporal terminations**	**Number mentions**	**References**
IFGop	ITG	6	Sarubbo et al. (2016)
			Martino et al. (2011)
			Barrick et al. (2006)
			Rojkova et al. (2016)
			Glasser and Rilling (2008)
			Yagmurlu et al. (2016)
	MTG	9	Vassal et al. (2016)
			Sarubbo et al. (2016)
			Barrick et al. (2006)
			Lawes et al. (2008)
			Rojkova et al. (2016)
			Zemmoura et al. (2014)
			Glasser and Rilling (2008)
			Fernández‐Miranda et al. (2015)
			Yagmurlu et al. (2016)
	STG	8	Vassal et al. (2016)
			Sarubbo et al. (2016)
			Barrick et al. (2006)
			Rojkova et al. (2016)
			Glasser and Rilling (2008)
			Thiebaut de Schotten et al. (2012)
			Fernández‐Miranda et al. (2015)
			Yagmurlu et al. (2016)
IFGtri	ITG	2	Martino et al. (2011)
			Glasser and Rilling (2008)
	MTG	5	Vassal et al. (2016)
			Martino et al. (2011)
			Zemmoura et al. (2014)
			Glasser and Rilling (2008)
			Yagmurlu et al. (2016)
	STG	3	Vassal et al. (2016)
			Thiebaut de Schotten et al. (2012)
			Yagmurlu et al. (2016)
IFG	ITG	2	Martino, Silva‐Freitas et al. (2013)
			Thiebaut de Schotten et al. (2012)
	MTG	5	Catani et al. (2005)
			Forkel et al. (2020)
			Martino, Silva‐Freitas et al. (2013)
			Glasser and Rilling (2008)
			Yagmurlu et al. (2016)
	STG	3	Catani et al. (2005)
			Forkel et al. (2020)
			Gharabaghi et al. (2009)
MFG	ITG	6	Sarubbo et al. (2016)
			Martino, Silva‐Freitas et al. (2013)
			Glasser and Rilling (2008)
			Thiebaut de Schotten et al. (2012)
			Fernández‐Miranda et al. (2015)
			Yagmurlu et al. (2016)
	MTG	7	Vassal et al. (2016)
			Sarubbo et al. (2016)
			Martino, Silva‐Freitas et al. (2013)
			Glasser and Rilling (2008)
			Thiebaut de Schotten et al. (2012)
			Fernández‐Miranda et al. (2015)
			Yagmurlu et al. (2016)
	STG	4	Frey et al. (2008)
			Vassal et al. (2016)
			Sarubbo et al. (2016)
			Gharabaghi et al. (2009)
vPrCG	ITG	7	Sarubbo et al. (2016)
			Martino et al. (2011)
			Martino, Silva‐Freitas et al. (2013)
			Glasser and Rilling (2008)
			Thiebaut de Schotten et al. (2012)
			Fernández‐Miranda et al. (2015)
			Yagmurlu et al. (2016)
	MTG	8	Vassal et al. (2016)
			Sarubbo et al. (2016)
			Martino et al. (2011)
			Martino, Silva‐Freitas et al. (2013)
			Glasser and Rilling (2008)
			Thiebaut de Schotten et al. (2012)
			Fernández‐Miranda et al. (2015)
			Yagmurlu et al. (2016)
	STG	4	Vassal et al. (2016)
			Sarubbo et al. (2016)
			Glasser and Rilling (2008)
			Yagmurlu et al. (2016)
PrCG	ITG	1	Barrick et al. (2006)
	MTG	3	Barrick et al. (2006)
			Lawes et al. (2008)
			Forkel et al. (2020)
	STG	2	Barrick et al. (2006)
			Forkel et al. (2020)

Abbreviations: IFG, inferior frontal gyrus; IFGop, pars opercularis; IFGtri, pars triangularis; MFG, middle frontal gyrus; PrCG, precentral gyrus; vPrCG ventral precentral gyrus; STG, superior temporal gyrus; MTG, middle temporal gyrus; ITG, inferior temporal gyrus.

For what concerns the functional counterpart, we adopt the exact same cumulative approach already illustrated for the structural description to present the functional literature about the AF. Indeed, one single bundle is very likely to support multiple cognitive processes by playing different roles in different networks (Forkel et al., 2021). Since to date there is no recent review paper that summarizes the functional literature of the AF, we sampled the experimental works testing the involvement of this fiber pathway in a defined function, using both direct techniques (i.e., direct electrical stimulation (DES)) and correlational neuroimaging‐behavioral measurements (see Table 2 for references), with the aim to highlight the main different cognitive domains whose integration is mediated by this bundle. For this purpose, we conducted a literature search in Google Scholar using a combination of the strings “arcuate fasciculus” and “function”, “cognitive functions”, “behavioral correlates”, “linguistic”, “non‐linguistic”, and again, as the research highlighted more specific domains, “verbal memory”, “social cognition”, “spatial cognition”, “music perception”. Those works that imply a putative functional role of the bundle as a consequence of their findings but with no direct testing were not included.

**TABLE 2:** List of the studies reviewed that outlined the functional involvement of the arcuate fasciculus (AF) in specific functional classes and the respective domains classified according to the present cumulative approach.

**Functional class**	**Domain**	**References**	**Specification**	**Hemisphere**
Language	Speech production	Bates et al. (2003)	Verbal fluency deficit	LH
		Hope et al. (2016)	Verbal fluency deficit	LH
		Ivanova et al. (2016)		LH
		Marchina et al. (2011)		LH
		Wang et al. (2013)		LH
	Speech comprehension	Geva et al. (2015)		LH
		Ivanova et al. (2016)		LH
		Wilson et al. (2011)		LH
	Pragmatics	Chen et al. (2018)	Speech tone perception	LH
		Geva et al. (2015)	Rhyme judgment	LH
		Glasser and Rilling (2008)	Prosody processing	RH
	Phonology	Bello et al. (2007)	Phonemic paraphasia	BI, RH only for left‐handed
		Benzagmout et al. (2007)	Phonemic paraphasia	LH
		Chan‐Seng et al. (2014)	Phonemic paraphasia	LH
		De Benedictis et al. (2014)	Phonemic paraphasia	LH
		Leclercq et al. (2010)	Phonemic paraphasia	BI, RH only for left‐handed
		Maldonado, Moritz‐Gasser, de Champfleur et al. (2011)	Phonemic paraphasia	LH
		Maldonado, Moritz‐Gasser, Duffau (2011)	Phonemic paraphasia	LH
		Sarubbo et al. (2016)	Phonemic paraphasia	LH
		Vassal et al. (2013)	Phonemic paraphasia	LH
		Saygin et al. (2013)	Phonological awareness	LH
		Vandermosten et al. (2012)	Phonological awareness	LH
		Yeatman et al. (2011)	Phonological awareness	LH
		Breier et al. (2008)	Repetition deficits	LH
		Catani et al. (2005)	Repetition deficits	LH
		Ellmore et al. (2009)	Repetition deficits	LH
		Fridriksson et al. (2010)	Repetition deficits	LH
		Geva et al. (2015)	Repetition deficits	LH
		Kümmerer et al. (2013)	Repetition deficits	LH
		Moritz‐Gasser and Duffau (2013)	Repetition deficits	LH
		Sierpowska et al. (2017)	Nonword repetition deficits	LH
		Janssen et al. (2023)	Nonword repetition	LH
		Saur et al. (2008)	Repetition	LH
		Glasser and Rilling (2008)		LH
		Mullen et al. (2011)		BI, developping children
		Phillips et al. (2011)		LH
		Sarubbo et al. (2015)		LH
		Sarubbo et al. (2020)		LH
	Syntax	Friederici (2009)		LH
		Griffiths et al. (2013)		LH
		Leclercq et al. (2010)		BI, RH only for left‐handed
		Papoutsi et al. (2011)		LH
		Wilson et al. (2011)		LH
	Semantics	Leclercq et al. (2010)	Semantic paraphasia	BI, RH only for left‐handed
		Teubner‐Rhodes et al. (2016)	Vocabulary knowledge	LH
		Janssen et al. (2023)	Verb generation	LH
		Zigiotto et al. (2022)		LH
		Glasser and Rilling (2008)		LH
	Articulatory	Leclercq et al. (2010)	Speech arrest	BI, RH only for left‐handed
	Word learning	López‐Barroso et al. (2013)		LH
	Naming	Bello et al. (2007)	Anomia	BI, RH only for left‐handed
		Duffau et al. (2002)	Anomia	LH
		Sarubbo et al. (2015)	Anomia	LH
		Geva et al. (2015)		LH
		Hope et al. (2016)		LH
		Marchina et al. (2011)		LH
		McDonald et al. (2008)		BI
		Moritz‐Gasser and Duffau (2013)		LH
	Reading	Saygin et al. (2013)		LH
		Yeatman et al. (2011)		LH
		Yeatman, Dougherty, Ben‐Shachar et al. (2012)		LH
		Yeatman, Dougherty, Myall et al. (2012)		LH
Verbal memory	Verbal memory	Reijmer et al. (2012)		LH
		McDonald et al. (2008)		BI
	Verbal working memory	Meyer et al. (2014)		LH
	Verbal short‐term memory	Papagno et al. (2017)	Digit span order errors	LH
Social cognition	Social cognition	Chiang et al. (2017)		RH
	False‐belief understanding	Grosse Wiesmann et al. (2017)	ToM development	BI, developping children
	Face‐based mentalizing	Yordanova et al. (2017)		RH
		Cabinio et al. (2015)		RH
		Herbet et al. (2014)		RH
		Nakajima et al. (2018)		RH
Music perception	Tone discrimination	Loui et al. (2009)		RH
	Pitch‐related grammar learning	Loui et al. (2011)		RH
	Rhythm modulation detection	Vaquero et al. (2021)		LH
Spatial cognition	Visuospatial attention	He et al. (2007)	Spatial neglect	RH
		Rolland et al. (2018)	Spatial neglect	RH
		Roux et al. (2011)	Spatial neglect	RH
		Umarova et al. (2010)	Change detection	RH
	Spatial perception	Sarubbo et al. (2015)		RH

Abbreviations: BI, bilateral; LH, left hemisphere; RH, right hemisphere.

In line with the renowned notion of the left and right AF contributing to different cognitive domains (Nakajima et al., 2019), we will report findings according to the hemisphere they are related to. Although most of the studies considered focused their investigation of AF functionality on a specific hemisphere, there are some works that carried out their analysis on the whole brain and were therefore able to relate different functions to this bundle depending on its hemispheric location, or, on the contrary, to specify that the same function is mediated by the AF from both hemispheres. Those studies that found the same function to be mediated by bilateral AF will be considered as one evidence for each hemisphere.

The frontal gyri innervated by the AF are the IFG, the precentral gyrus (PrCG) and the middle frontal gyrus (MFG), reported in decreasing order based on how many times connections to these areas have been described. When addressing the anterior terminations of the AF in the PrCG many authors specified that only the most ventral part of this area is reached by the AF. Similarly, for what concerns the IFG, different works refer to terminations in IFGop, in IFGtri or in the whole gyrus. On the other hand, the temporal region which has more often been described to be connected by the AF is the middle temporal gyrus (MTG), followed by the inferior (ITG) and the superior (STG) temporal gyri.

Overall, all the cortical areas reported above seem to be mutually interconnected, but there is a great variability in how many works described the existence of defined wiring patterns. The connections that have more commonly been described as part of the AF run between the various gyri of the frontal cortex and the MTG: most of the reviewed studies describe IFGop–MTG connectivity, whereas connections between this temporal area and the vPrCG and the MFG have less frequently been reported. Although connections between IFGop and the STG seem to represent a typical layout of AF's wiring in the works we sampled, less of them report connections linking both the vPrCG and the MFG to the STG or to the ITG. On the contrary, less descriptions of connections from the IFGop to the ITG have been reported compared to connectivity between this gyrus and the STG. As mentioned in the previous section, few works among the ones we considered found anterior terminations of the AF in IFGtri. Still, the reported involvement of this region reflects the proportion of descriptions made for IFGop, with less mentions of connectivity from IFGtri to MTG, STG, and ITG, respectively. The same applies to those works that referred to IFG in its entirety; however, among those that described AF connectivity to the PrCG by considering the whole gyrus, a description of connections running from this area to the MTG, the STG, or to the ITG are also reported, in slight countertrend compared to the number of authors that specified the involvement of the sole ventral part of this region. The structural connections and the list of studies considered are reported in Table 1.

**FIGURE 3:**
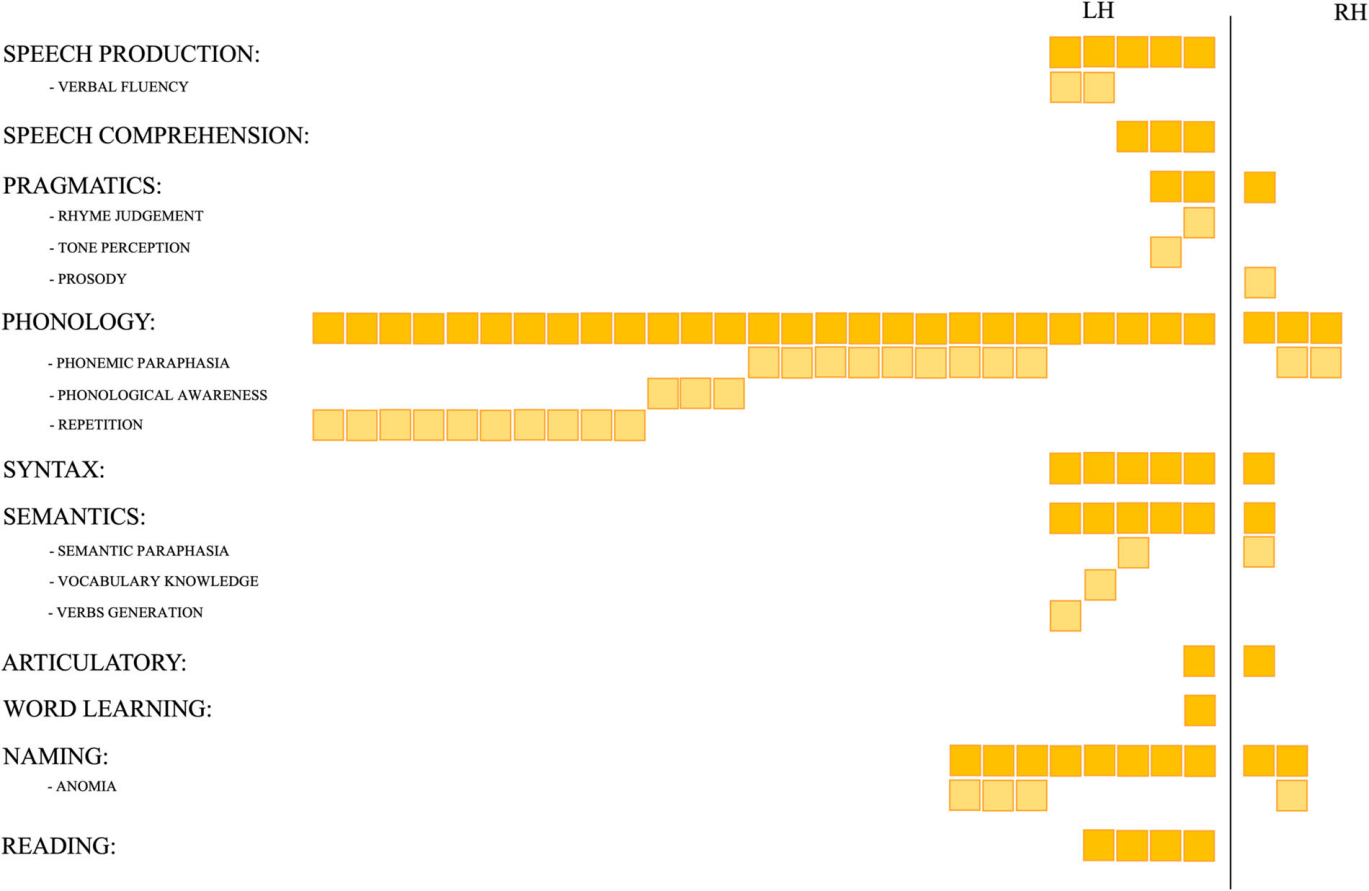
Schematic cumulative representation of the number of studies that described the role of the arcuate fasciculus (AF) in mediating linguistic abilities. All the revised works have been classified depending on the general ability, linguistic domain, or specific language‐related skills that they tackle (see text for a more detailed description). Each solid‐colored square represents one account for the defined category; shaded squares have been added below each of these broad categories in the case of studies specifying a precise subcategory (being it a particular deficit related to AF damage or a defined ability correlating with its features).

**FIGURE 4:**
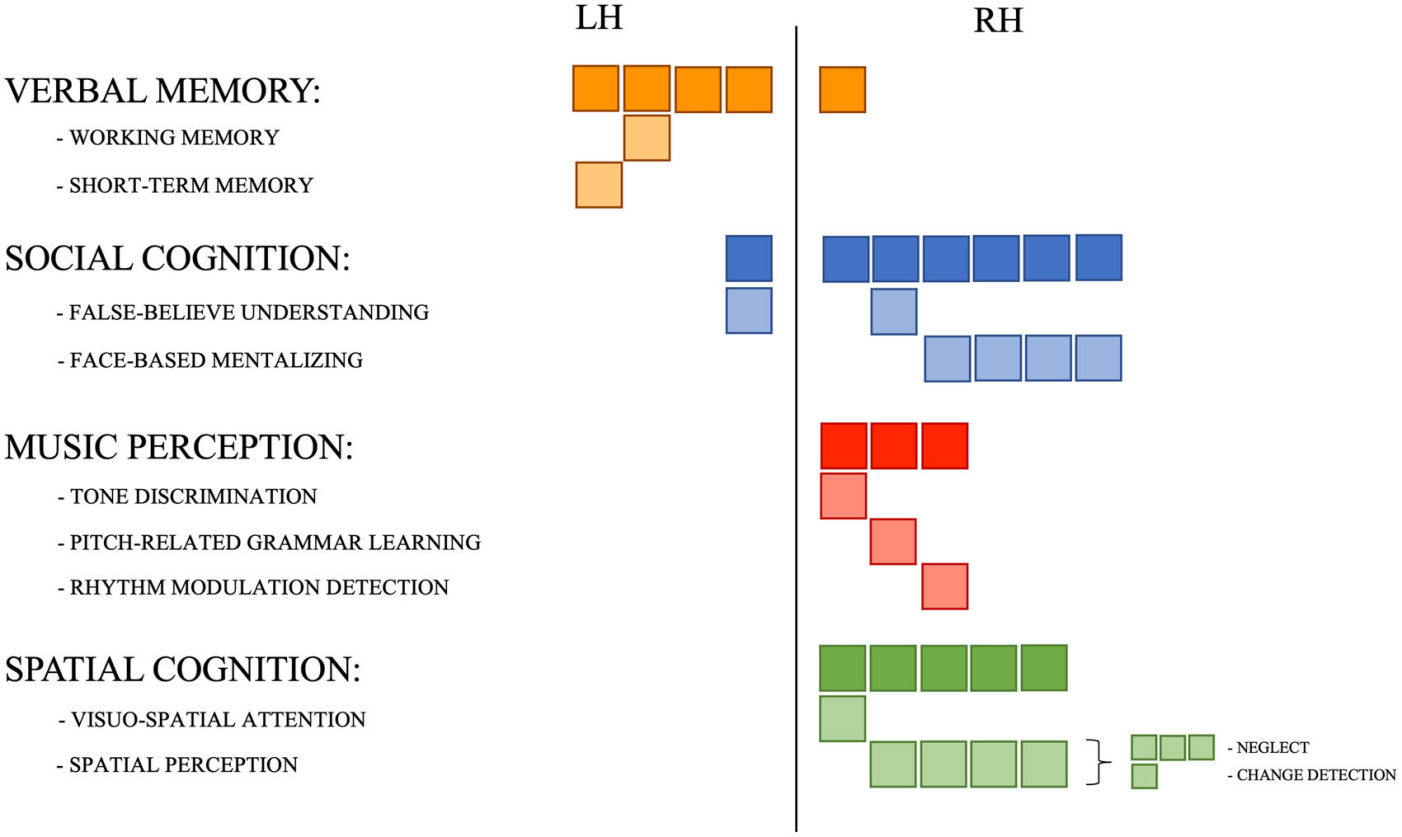
Schematic cumulative representation of the number of studies that described the role of the arcuate fasciculus (AF) in mediating nonlinguistic abilities. Each solid‐colored square represents one account for the defined category; shaded squares have been added below each of these broad categories in the case of studies specifying a precise subcategory (being it a particular deficit related to AF damage or a defined ability correlating with its features).

As we described in the historical section, the AF has been implied in linguistic functions since its first characterizations (Broca, 1865; Geschwind, 1965; Wernicke, 1874). This initial assumption based on anatomo‐clinical correlation studies was validated with the advent of functional MRI, intraoperative mapping with DES, and correlational studies of tractography‐derived bundles’ parameters with behavioral testing. Indeed, 41 out of the 59 studies we hereby reviewed support the involvement of direct dorsal frontotemporal connections in the linguistic domain. The congregate analysis of these works enlightens the complexity of language as a multifaceted ability, composed of several components whose coordinated interplay gives rise to more general abilities. Given this complexity and the magnitude of the number of works specifically investigating the role of the AF in language, findings regarding this function and nonlinguistic cognitive domains will be presented separately (Figure 3 for linguistic abilities and Figure 4 for the other domains, for references see Table 2).

Different works among the ones we surveyed generally stated a relationship between structural features of the left AF, such as the degree of tract lesioning or the decrease of its microstructural diffusivity in pathology, and a general impairment of speech production and comprehension. Still, most of them focused on specific aspects of language, expressed both in terms of a defined cognitive impairment associated with a lesion or in terms of associations between structural/functional and behavioral evidence in healthy participants. These instances can be summarized considering four out of the five domains of language (i.e., pragmatics, phonology, syntax, and semantics). Although only few of the works reviewed reported the involvement of the bilateral AF in functions related to the pragmatics domain—the left hemisphere bundle was related to rhyme judgment and speech tone perception, and the right one was proposed to support prosody processing—most of them stressed an association between this WM pathway in the left hemisphere and phonological functions. Among these, studies using DES mapping described phonemic paraphasia in correspondence of AF stimulation; studies conducted both on healthy participants and with intraoperative stimulation in patients reported an association with repetition abilities, whereas studies conducted on a normative population stressed the role of the AF in phonological awareness, a predictor of reading skills. Moreover, the left AF is reported to play a role in the syntactic domain and in semantic aspects of language. In particular, one of the studies considered reported an association between the bundle's microstructural tractography‐derived measures and vocabulary knowledge, whereas another investigation with DES mapping observed semantic paraphasia following AF stimulation.

Up to now, we discussed the involvement of the AF in linguistic domains‐related functions mainly in the left hemisphere. Nevertheless, we also identified some reports of linguistic functions being mediated by the right AF (see Figure 3). The evident imbalance between the number of descriptions of an association between left and right AF and language is in line with the notorious strong left‐lateralization of this function in the normative population (Frost, 1999; Malik‐Moraleda et al., 2022; Wang et al., 2019). Moreover, it is worth considering that all the reports herein reviewed that attribute linguistic abilities to the right AF are studies relating a bilateral activation for that function. If we regard these works more carefully, it can be inferred that the relationship of the right AF with phonemic paraphasia, general syntactic abilities, and semantic paraphasia is found in left‐handed patients for which language is more likely to be lateralized in the right hemisphere compared to the general population (Szaflarski et al., 2002). The only non‐neurosurgical description of a correlation between this bundle's tractography‐derived measurements in the right hemisphere and phonological abilities was conducted on a cohort of developing children, for whom the fine wiring of linguistic functional networks might still be in progress (Holland et al., 2007).

In the collection of studies we reviewed, there are some other linguistic abilities that, although not being domain‐specific but rather arising from the interplay of multiple macrodomains, have been found to be modulated by the AF, namely, word learning and the development of reading abilities. Finally, a very complex ability that relies on different linguistic domains, that is, naming, has been defined to be supported by the AF mainly in the left hemisphere: indeed, few studies report anomia after AF stimulation during DES subcortical mapping.

Although it is evident that the left AF mainly mediates linguistic abilities, our review revealed an involvement of this bundle also in verbal memory. However, out of this sample, one work focuses its definition of verbal memory on working memory, whereas one other study specifies that the left AF might be involved in verbal short‐term memory since its direct stimulation during surgery causes item errors during a digit span task. Another cognitive function that has been attributed to the AF is social cognition, with a role in false‐belief understanding in developing children (bilateral AF) and in face‐based mentalizing (right AF). According to the review we carried out, there are two other nonlinguistic cognitive functions that have been attributed to the right AF: music perception (tone discrimination and grammar learning in the domain of pitch as well as rhythm modulation detection) and spatial cognition (spatial perception and visuospatial attention). The full lists of studies, separated according to specific functions, are reported in Table 2.

## 3 AF TOPOGRAPHICAL ANATOMY AND SURGICAL IMPLICATIONS

Our literature review upholds the intricacy of the AF structural wiring as the foundation of a likewise considerable functional potentiality. Despite WM pathways have themselves limited structural plastic potential once connections are established, the extension of this bundle constitutes the anatomical substrate of a possibly enormous postlesional functional reorganization (De Benedictis & Duffau, 2011; Duffau, 2009, 2014, 2018). Being it a mediator of the communication between frontal and temporal lobes by crossing the parietal one, thorough knowledge of this bundle is mandatory to plan and perform safe surgeries involving all these regions, to avoid severe postsurgical sequelae. Indeed, a recent work contains considerable reports of transient or permanent language deficits (in 11.7%–54.5% and 1.7%–18.2% of cases, respectively) for tumors’ resections involving these cortices (Fang et al., 2021). In particular, the worst language prognosis was reported for lesions located around the postcentral (PoCG) and the supramarginal (SMG) gyri, that correspond, in fact, to the segment of the AF course with the highest fiber density, and therefore the lowest compensating capacity in respect to its anterior and posterior portions (Fang et al., 2021; Herbet et al., 2016; Plaza et al., 2009; van Geemen et al., 2014). Moreover, and as previously mentioned, disconnection of the right AF has been related to impairment in face‐based mentalizing (Herbet et al., 2014; Yordanova et al., 2017).

Consequently, a careful and patient‐tailored surgical planning for the preservation of eloquent subcortical structures, including the AF, is of foremost importance. This last section reports four different surgical cases of glioma resection involving the AF territories. Each of them addresses not only how the functional information collected during the procedure drives the definition of the resection boundaries but also how knowledge about the AF possible extension and functional implications drives surgical planning. This envisages (i) an accurate preoperative neuropsychological assessment for the selection of the most adequate tasks to be administered during systematic cortical and subcortical electrical mapping in awake conditions based on the location of the lesion (Bu et al., 2021; Martino, Gomez et al., 2013; Peraud et al., 2004; Rolland et al., 2018; Sarubbo et al., 2020); (ii) the evaluation of which WM pathways will possibly be impacted by the specific procedure, coupled with a tractographic 3D reconstruction of the WM bundles of interest.

For what concerns intraoperative neuropsychological assessment, the denomination task is normally used for procedures concerning the AF territories within the left language‐dominant hemisphere to test the emergence of phonemic paraphasia or pure anomia with DES. Electrostimulation of the right AF can evoke transient mentalizing troubles or spatial neglect: The Reading the Mind in the Eyes (Baron‐Cohen et al., 2001; Herbet et al., 2014; Sarubbo et al., 2020; Vigneau et al., 2006; Yordanova et al., 2017) and the line bisection tasks (Bartolomeo et al., 2007; He et al., 2007; Rolland et al., 2018; Roux et al., 2011; Sarubbo et al., 2015, 2020; Thiebaut de Schotten, 2005) are the preferential neuropsychological evaluation tools adopted in this case. In general, intraoperative cognitive testing gives real‐time feedback to the operator about the functional relevance of a given structure and, therefore, represents a precious directive on whether it is safe to resect it.

**FIGURE 5:**
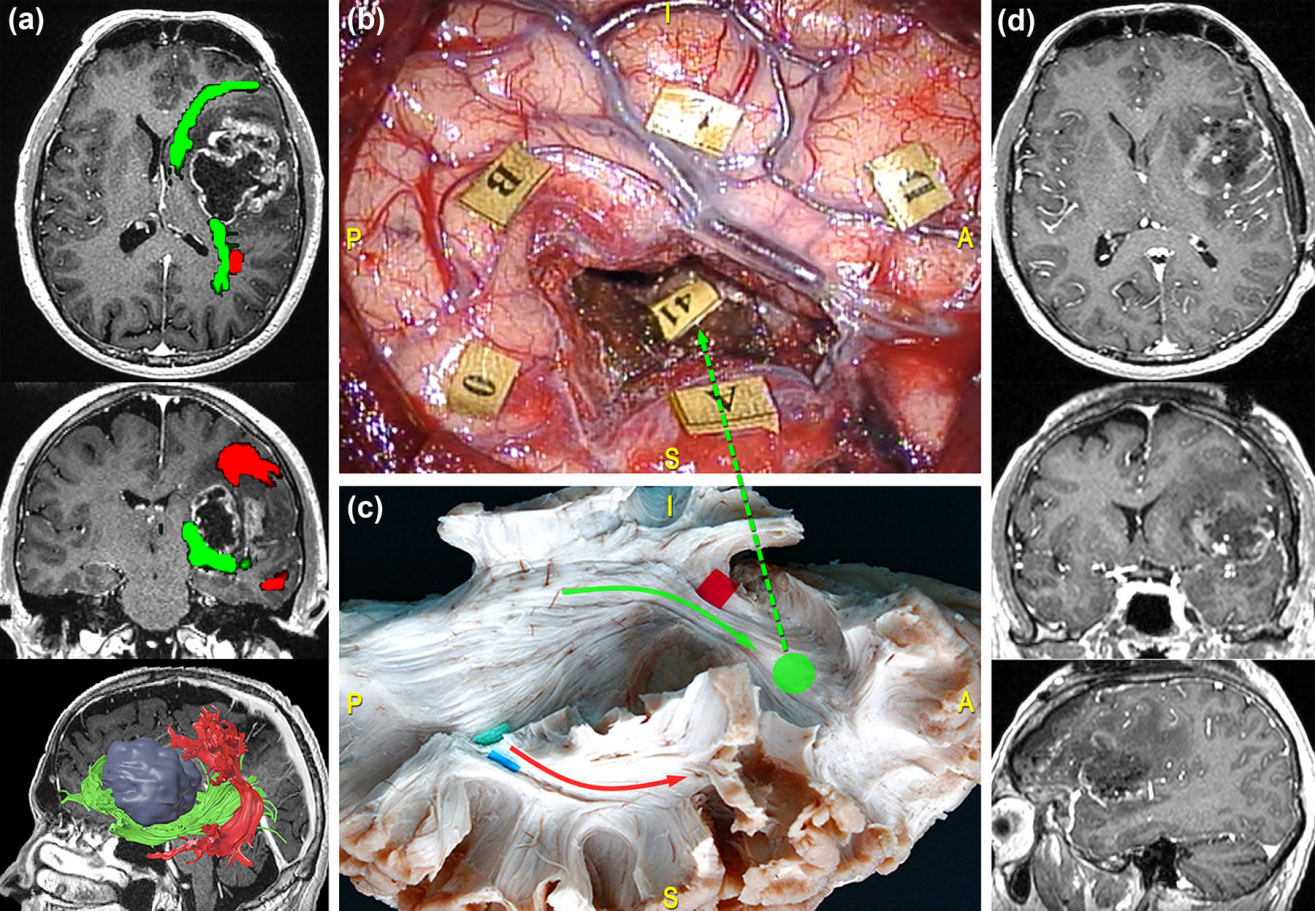
Surgical case concerning a 42‐year‐old man who underwent resection of a high‐grade glioma located within the left dominant fronto‐insular region. (a) Preoperative magnetic resonance imaging (MRI) (from top to bottom: axial, coronal, and 3D‐sagittal sequences) combined with tractographic reconstruction of the left arcuate fasciculus (AF) (red) and inferior fronto‐occipital fasciculus (IFOF) (green). (b) Intraoperative picture showing the tumor's resection performed with “asleep‐awake‐asleep” technique. Tag 0 refers to speech arrest site at the cortical level, while stimulation in correspondence of tag 41, representing IFOF's frontal projections elicited anomia, semantic paraphasia, and perseveration during denomination task. (c) Dissection of the perisylvian region with Klingler technique. The specimen has been oriented according to the surgical perspective. The green circle corresponds to the site of direct electrical stimulation (DES) stimulation along the course of the frontal projections of the IFOF (green arrow). (d) Postoperative MRI showing the resection on the axial, coronal, and sagittal plane from top to bottom.

As briefly mentioned above, different surgical procedures require to carefully consider the patient's AF wiring and the related functional networks. For instance, when approaching a tumor located in the frontal lobe, namely, under the IFG, the MFG, and the dorsolateral prefrontal cortex, it should be considered that the AF courses horizontally at this level, and that its fibers are strongly intertwined with the deep layer of the inferior fronto‐occipital fasciculus (IFOF), which locally follows a vertical temporo‐frontal orientation. In this case, the fibers of the AF that extend to the IFG represent the deep and posterior functional boundary. At this level, AF terminations overlap with the most ventral segment of the superior longitudinal fasciculus (SLF III) and with the superficial layer of the IFOF (De Benedictis et al., 2012; Duffau, 2018; Dziedzic et al., 2022). Figure 5 illustrates the case of a 42‐year‐old man, who underwent resection of a high‐grade glioma located within the left dominant fronto‐insular region. Although cortical DES in awake condition allowed to identify one site eliciting speech arrest when stimulated, subcortical mapping during resection revealed eloquent functional sites evoking anomia, semantic paraphasia, and perseveration during denomination task, in correspondence to the IFOF's frontal projections.

When dealing with insular tumors, many studies support the safety and reliability of the transopercular approach, although it requires passing through IFGtri and IFGop (classically known as Broca's area). For this type of procedure, the AF constitutes the deep, superior, and posterior functional limit of resection (Dziedzic et al., 2022).

**FIGURE 6:**
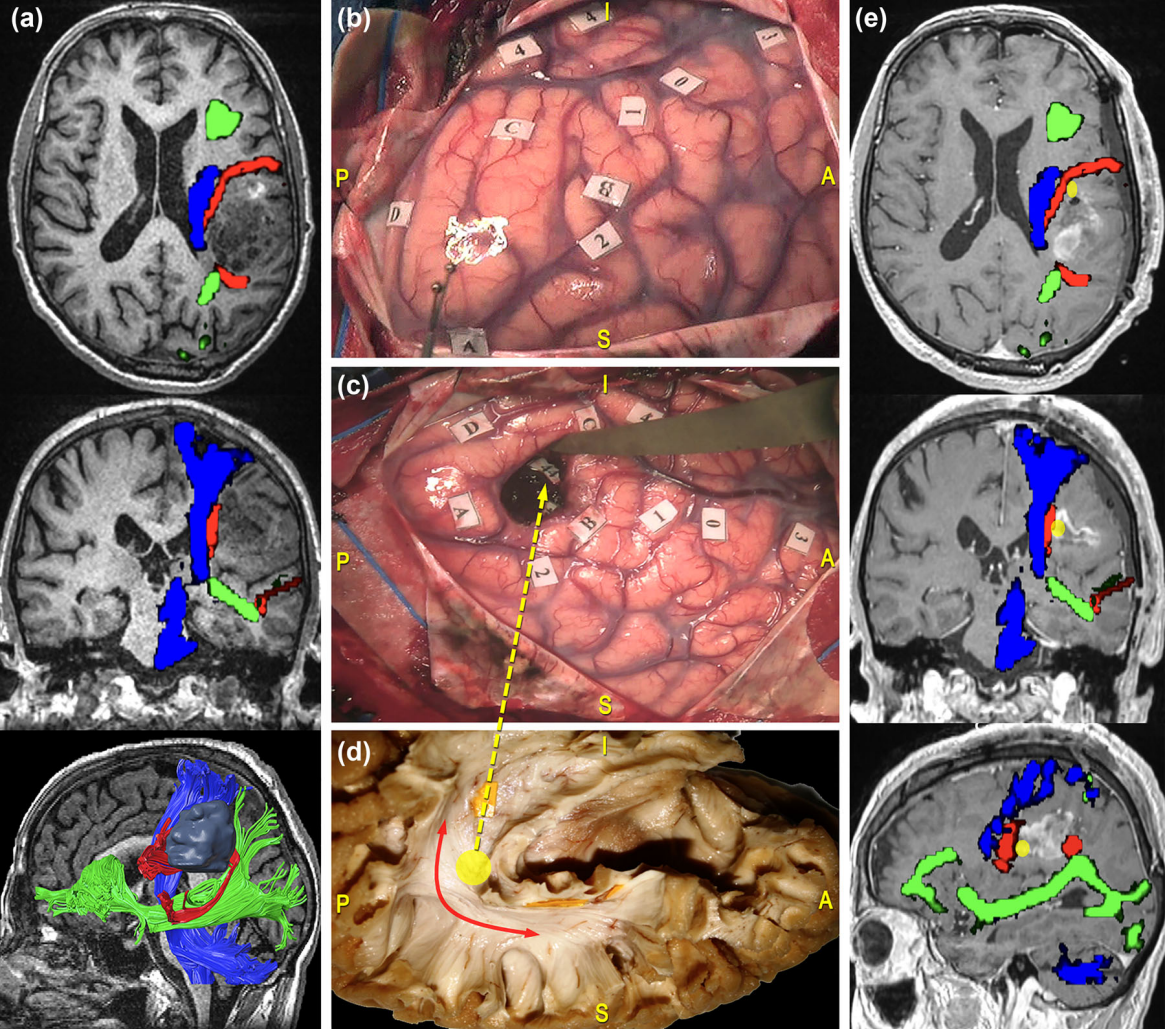
Surgical case concerning a 69‐year‐old man who underwent resection of a high‐grade glioma located within the left dominant frontoparietal region. (a) Preoperative magnetic resonance imaging (MRI) (from top to bottom: axial, coronal, and 3D‐sagittal sequences) combined with tractographic reconstruction of the left arcuate fasciculus (AF) (red), the inferior fronto‐occipital fasciculus (IFOF) (green) and the corticospinal tract (CST) (blue). (b) Intraoperative picture showing the cortical mapping performed in awake condition with direct electrical stimulation (DES). Functional responses were found at the level of the precentral gyrus (PrCG), eliciting facial, and arm contractions (tag 1 and 2); of the ventral premotor cortex (VPMC), eliciting speech arrest (tag 0); of the pars triangularis (IFGtri), eliciting anomia (tag 3); of the posterior third of the superior temporal gyrus (STG), eliciting phonemic paraphasia (tag 4). (c) Intraoperative picture showing the results of subcortical mapping. Phonemic paraphasia was elicited at the level of the AF (tag 44). (d) Anatomical specimen of a left hemisphere oriented according to the surgical perspective and showing the AF course corresponding to the stimulation site (yellow circle and arrow) (e) Postoperative MRI (from top to bottom: axial, coronal, and sagittal sequences) combined with tractographic reconstruction of the left AF (red), the IFOF (green), and the CST (blue). The yellow circle corresponds to the site of elicitation of phonemic paraphasia (tag 44), matching with the AF course.

**FIGURE 7:**
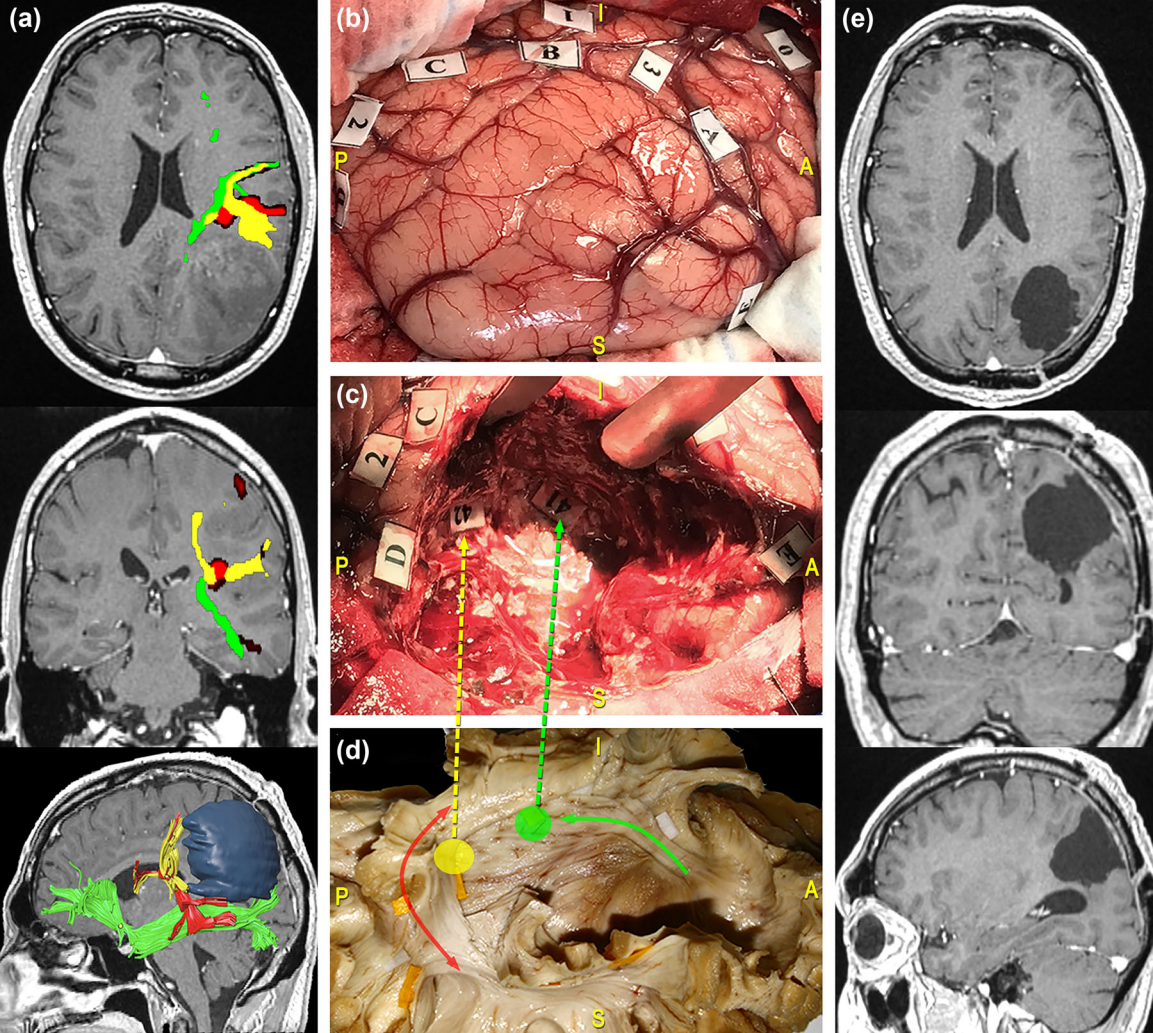
Surgical case concerning a 41‐year‐old man who underwent resection of a high‐grade glioma located within the superior parietal lobule of the left dominant hemisphere. (a) Preoperative magnetic resonance imaging (MRI) (from top to bottom: axial, coronal, and 3D‐sagittal sequences) combined with tractographic reconstruction of the left arcuate fasciculus (AF) (red), the inferior fronto‐occipital fasciculus (IFOF) (green), and the temporoparietal component of the superior longitudinal fasciculus (SLF) (yellow). (b) Intraoperative picture showing the tumor's resection performed according to the “asleep‐awake‐asleep” protocol. Direct electrical stimulation (DES) allowed to identify eloquent cortical sites including: the postcentral gyrus (PoCG), eliciting right facial paresthesia when stimulated (tag 0); the supramarginal gyrus (SMG), eliciting speech arrest (tag 1) and anomia (tag 2 and 3) during naming tasks. (c) During tumor resection, subcortical mapping allowed to evoke semantic paraphasia at the level of the dorsal part of the IFOF (tag 41) and verbal apraxia during the denomination task, along the temporoparietal component of the SLF and the AF (tag 42). (d) Dissection of the perisylvian region with Klingler technique. The specimen has been oriented according to the surgical perspective. The colored tags correspond to the IFOF (green circle and arrow) and the temporoparietal SLF/AF (yellow circle, red, and yellow arrows). Postoperative MRI shows complete tumor resection.

At the level of the temporo‐parieto‐occipital junction, the AF participates in a complex system of connections involving many eloquent pathways, and it is therefore relevant in the case of lesions involving the inferior parietal lobule. Indeed, at this level, the AF forms a compact vertical stem that runs deep and parallel to the temporoparietal component of the SLF, crossing even deeper tracts characterized by a horizontal anterior‐to‐posterior course, namely, the middle and inferior longitudinal fasciculi (MdLF and ILF), the IFOF, and the optic radiation (Martino et al., 2011; Sarubbo et al., 2016). Here, the AF is located at the anterolateral wall of the cavity (De Benedictis et al., 2014). Figure 6 reports the case of a 69‐year‐old man, who underwent resection of a high‐grade glioma located within the left dominant frontoparietal region. Functional responses were found at the cortical level when stimulating the PrCG, eliciting facial and arm contractions, the ventral premotor cortex (VPMC), eliciting speech arrest, the IFGtri, eliciting anomia, and the posterior third of the STG, eliciting phonemic paraphasia. No functional responses were found during stimulation of the superior parietal lobule. On the other hand, subcortical mapping in correspondence of the AF was related to phonemic paraphasia. Figure 7 shows the case of a 41‐year‐old man, who underwent resection of a high‐grade glioma located within the superior parietal lobule of the left dominant hemisphere. DES allowed to identify eloquent cortical sites, including the PoCG, eliciting right facial paresthesia when stimulated, and the SMG, related to speech arrest and anomia during picture naming task. No functional response was found for neither motor nor visuospatial tasks in the left parietal lobule. During tumor resection, subcortical mapping evoked semantic paraphasia along the dorsal component of the IFOF and verbal apraxia during the denomination task in correspondence of the temporoparietal component of the SLF and the AF.

**FIGURE 8:**
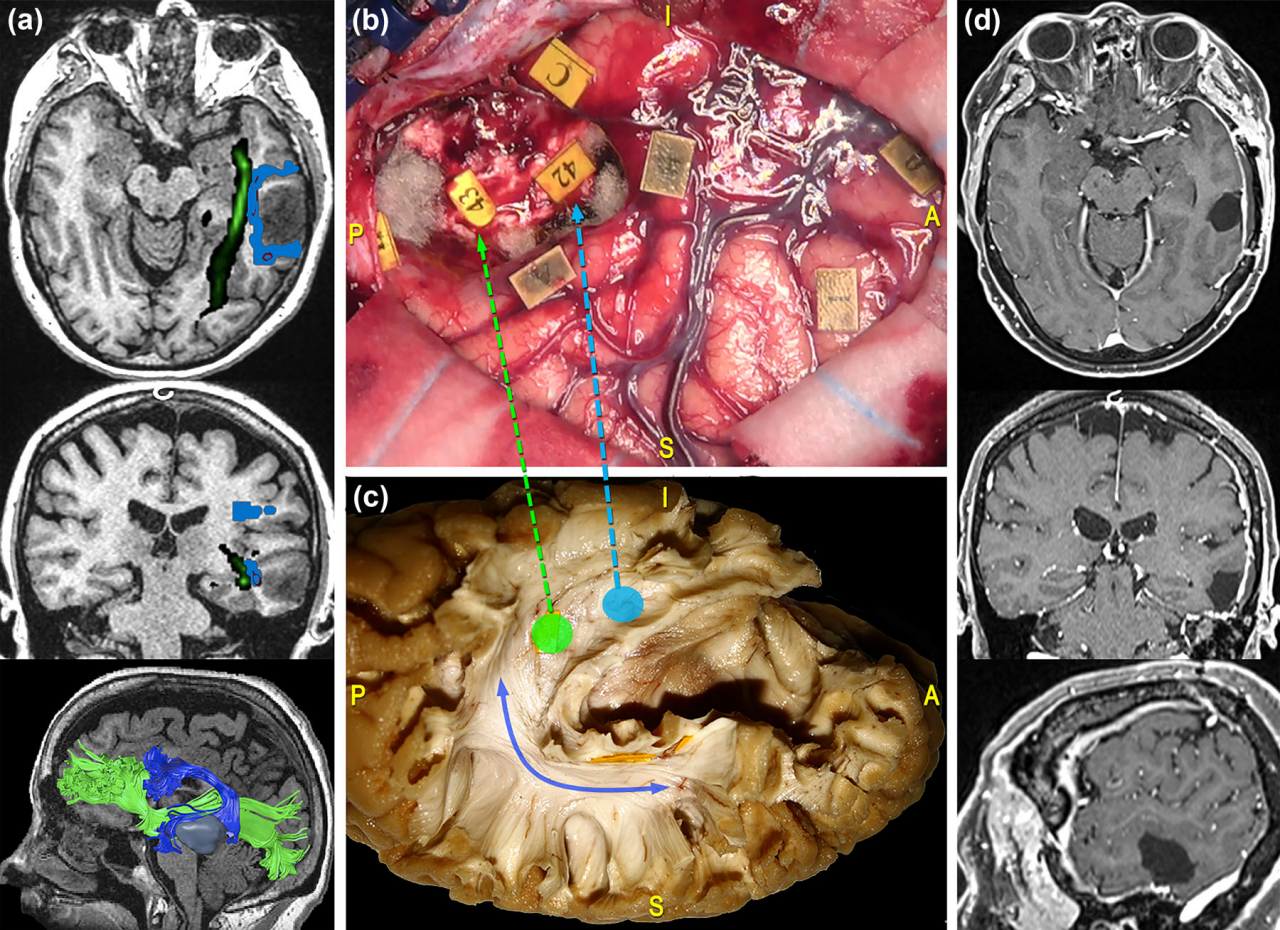
Surgical case concerning a 47‐year‐old woman who underwent resection of a high‐grade glioma located within the posterior third of the middle temporal gyrus (MTG) of the left dominant hemisphere. (a) Preoperative magnetic resonance imaging (MRI) (from top to bottom: axial, coronal, and 3D‐sagittal sequences) combined with tractographic reconstruction of the left arcuate fasciculus (AF) (blue) and inferior fronto‐occipital fasciculus (IFOF) (green). (b) Intraoperative picture showing the tumor's resection performed with “asleep‐awake‐asleep” technique. Direct electrical stimulation (DES) in awake condition allowed to identify eloquent cortical sites, including the ventral premotor cortex (VPMC), eliciting speech arrest (tag 0), and the ventral motor strip of the face (tag 1). During tumor's resection, the subcortical mapping allowed to identify the resection boundaries. These were established by eliciting phonemic paraphasia during the denomination task at the level of the anteroinferior portion of the AF (tag 42) and sematic paraphasia at the level of the IFOF (tag 43). (c) Dissection of the perisylvian region with Klingler technique. The specimen has been oriented according to the surgical perspective. The blue line corresponds to the AF stem and the light‐blue and green circles and arrows to the site where the IFOF and the AF were stimulated, respectively. (d) Postoperative MRI showed the complete tumor resection on the axial, coronal, and sagittal planes from top to bottom.

The AF also represents an important functional limit when performing a temporal lobectomy for tumor removal or epilepsy surgery, especially in the dominant hemisphere. In these cases, the AF temporal terminations constitute the subcortical posterolateral boundary of resection (Duffau et al., 2008). When approaching the ventral left temporal region, corresponding to the visual word form area, DES mapping is recommended to differentiate the course of the AF from the one of the ILF: Stimulation of the left AF at this level would induce pure anomia by interfering with the integration of visual information into the language network (Duffau et al., 2008; Mandonnet et al., 2017; Sarubbo et al., 2020). At this level, also the course of the IFOF should be considered. Figure 8 illustrates the case of a 47‐year‐old woman who underwent resection of a high‐grade glioma located within the posterior third of the MTG of the left dominant hemisphere. DES in awake condition allowed to identify the ventral motor strip of the face, and cortical stimulation of the VPMC elicited speech arrest. No functional response was found at denomination, reading comprehension and motor tasks during stimulation of the middle and posterior parts of the temporal region. Subcortical mapping allowed to identify the resection boundaries represented by the anteroinferior portion of the AF, that once stimulated led to phonemic paraphasia during denomination task, and the IFOF, whose stimulation led to semantic paraphasia.

Finally, the AF is strictly related to the ventricular system. Indeed, the superior part of the AF runs lateral to the superior two thirds of the frontal horn and to the body of the lateral ventricle; its central segment runs lateral to the anterior two thirds of the atrium, whereas its inferior portion runs from the level of the posterior insular point (i.e., the junction between the inferior and the superior insular sulci) to the anterior tip of the temporal horn (Güngör et al., 2017). The understanding of these anatomical relationships is crucial to perform safe accesses to ventricular tumors, in particular when adopting an anterior frontal transcortical, posterior transcortical, or posterior transtemporal approach (De Benedictis et al., 2022).

## 4 CONCLUSIONS

The name *“arcuate fasciculus”* encloses two Centuries of neuroanatomical discoveries and evolutions. Since its first mention in Karl Friedrich Burdach's work (Burdach, 1822), this nomenclature became a pillar for the definition of the human brain's WM, and the associated knowledge increased concurrently with the methodological advancements of the past years. The investigations conducted on the AF concurred that this bundle is a crucial structural pathway for the integration of cognitive functions. Its shape, the large territories it connects, and the critical course it follows—interfacing with eloquent portions of the Superior and Inferior Longitudinal Systems as well as with projection fibers—make it one of the most critical association WM structures that require monitoring during surgical procedures. On the functional counterpart, many works demonstrate that the AF is critical for cognitive processing beyond language on both left and right sides. Indeed, this property can only be featured by a long‐range associative bundle connecting multimodal and highly distributed cortical areas, and therefore subserving the integration of different networks.

Given these premises, extensive knowledge about the AF structural extension, its relationship with other nearby WM structures, and the consequent implication in cognitive processes is essential to plan safe surgical approaches, and to consider all the variables that could affect the patient's outcome. We therefore summarized and reported multimodal evidence accounting for the current definition of the overall wiring and the functional relevance of the AF. The additional description of surgical cases of tumor resection considers how this knowledge can be translated into the surgical practice and describes practical tools and technical nuances for planning and performing safe resections with direct mapping of this critical structure.

## CONFLICT OF INTEREST STATEMENT

The authors declare no conflict of interest.

### PEER REVIEW

The peer review history for this article is available at https://publons.com/publon/10.1002/brb3.3107

## Data Availability

The data that support the findings of this study are available on request from the corresponding author. The data are not publicly available due to privacy or ethical restrictions.

## References

[brb33107-bib-0001] Agrawal, A. , Kapfhammer, J. P. , Kress, A. , Wichers, H. , Deep, A. , Feindel, W. , Sonntag, V. K. H. , Spetzler, R. F. , & Preul, M. C. (2011). Josef Klinglerʼs models of white matter tracts: Influences on neuroanatomy, neurosurgery, and neuroimaging. Neurosurgery, 69(2), 238–254. 10.1227/NEU.0b013e318214ab79 21368687

[brb33107-bib-0002] Baron‐Cohen, S. , Wheelwright, S. , Hill, J. , Raste, Y. , & Plumb, I. (2001). The “Reading the Mind in the Eyes” test revised version: A study with normal adults, and adults with Asperger syndrome or high‐functioning autism. Journal of Child Psychology and Psychiatry, 42(2), 241–251. 10.1111/1469-7610.00715 11280420

[brb33107-bib-0003] Barrick, T. R. , Lawes, I. N. , Mackay, C. E. , & Clark, C. A. (2006). White matter pathway asymmetry underlies functional lateralization. Cerebral Cortex, 17(3), 591–598. 10.1093/cercor/bhk004 16627859

[brb33107-bib-0004] Bartolomeo, P. , Thiebaut de Schotten, M. T. , & Duffau, H. (2007). Mapping of visuospatial functions during brain surgery. Neurosurgery, 61(6), E1340–E1340. 10.1227/01.neu.0000306126.46657.79 18162882

[brb33107-bib-0005] Basser, P. J. , Pajevic, S. , Pierpaoli, C. , Duda, J. , & Aldroubi, A. (2000). In vivo fiber tractography using DT‐MRI data. Magnetic Resonance in Medicine, 44, 625–632. 11025519 10.1002/1522-2594(200010)44:4<625::aid-mrm17>3.0.co;2-o

[brb33107-bib-0006] Bates, E. , Wilson, S. M. , Saygin, A. P. , Dick, F. , Sereno, M. I. , Knight, R. T. , & Dronkers, N. F. (2003). Voxel‐based lesion–symptom mapping. Nature Neuroscience, 6(5), 3. 12704393 10.1038/nn1050

[brb33107-bib-0007] Becker, Y. , Loh, K. K. , Coulon, O. , & Meguerditchian, A. (2022). The arcuate fasciculus and language origins: Disentangling existing conceptions that influence evolutionary accounts. Neuroscience & Biobehavioral Reviews, 134, 104490. 10.1016/j.neubiorev.2021.12.013 34914937

[brb33107-bib-0008] Benzagmout, M. , Gatignol, P. , & Duffau, H. (2007). Resection of world health orhanization grade II gliomas involving Broca's area: Methodological and functional considerations. Neurosurgery, 61(4), 13. 10.1227/01.NEU.0000298902.69473.77 17986935

[brb33107-bib-0009] Breier, J. I. , Hasan, K. M. , Zhang, W. , Men, D. , & Papanicolaou, A. C. (2008). Language dysfunction after stroke and damage to white matter tracts evaluated using diffusion tensor imaging. American Journal of Neuroradiology, 29(3), 483–487. 10.3174/ajnr.A0846 18039757 PMC3073452

[brb33107-bib-0010] Broca, P. (1865). Sur le siège de la faculté du langage articulé. Bulletins et Mémoires de La Société d'Anthropologie de Paris, 6, 337–393.

[brb33107-bib-0011] Bu, L.‐H. , Zhang, J. , Lu, J.‐F. , & Wu, J.‐S. (2021). Glioma surgery with awake language mapping versus generalized anesthesia: A systematic review. Neurosurgical Review, 44(4), 1997–2011. 10.1007/s10143-020-01418-9 33089447

[brb33107-bib-0012] Bunevicius, A. , Tamasauskas, S. , Deltuva, V. , Tamasauskas, A. , Radziunas, A. , & Bunevicius, R. (2014). Predictors of health‐related quality of life in neurosurgical brain tumor patients: Focus on patient‐centered perspective. Acta Neurochirurgica, 156(2), 367–374. 10.1007/s00701-013-1930-7 24254135

[brb33107-bib-0013] Burdach, K. F. (1822). Vom Baue und Leben des Gehirns (Vol. 2). Dyk.

[brb33107-bib-0014] Burdach, K. F. (1826). Vom Baue und Leben des Gehirns (Vol. 3). Dyk.

[brb33107-bib-0015] Cabinio, M. , Rossetto, F. , Blasi, V. , Savazzi, F. , Castelli, I. , Massaro, D. , Valle, A. , Nemni, R. , Clerici, M. , Marchetti, A. , & Baglio, F. (2015). Mind‐reading ability and structural connectivity changes in aging. Frontiers in Psychology, 6, 1808. 10.3389/fpsyg.2015.01808 26635702 PMC4659903

[brb33107-bib-0016] Catani, M. , Howard, R. J. , Pajevic, S. , & Jones, D. K. (2002). Virtual in vivo interactive dissection of white matter fasciculi in the human brain. Neuroimage, 17(1), 77–94. 10.1006/nimg.2002.1136 12482069

[brb33107-bib-0017] Catani, M. , Jones, D. K. , & ffytche, D. H. (2005). Perisylvian language networks of the human brain. Annals of Neurology, 57(1), 8–16. 10.1002/ana.20319 15597383

[brb33107-bib-0018] Chan‐Seng, E. , Moritz‐Gasser, S. , & Duffau, H. (2014). Awake mapping for low‐grade gliomas involving the left sagittal stratum: Anatomofunctional and surgical considerations. Journal of Neurosurgery, 120(5), 1069–1077. 10.3171/2014.1.JNS132015 24484222

[brb33107-bib-0019] Chen, X. , Zhao, Y. , Zhong, S. , Cui, Z. , Li, J. , Gong, G. , Dong, Q. , & Nan, Y. (2018). The lateralized arcuate fasciculus in developmental pitch disorders among mandarin amusics: Left for speech and right for music. Brain Structure and Function, 223, 2013–2024. 10.1007/s00429-018-1608-2 29322239

[brb33107-bib-0020] Chiang, H. , Chen, Y. , Lin, H. , Tseng, W. I. , & Gau, S. S. (2017). Disorder‐specific alteration in white matter structural property in adults with autism spectrum disorder relative to adults with ADHD and adult controls. Human Brain Mapping, 38(1), 384–395. 10.1002/hbm.23367 27630075 PMC6866870

[brb33107-bib-0021] De Benedictis, A. , & Duffau, H. (2011). Brain hodotopy: From esoteric concept to practical surgical applications. Neurosurgery, 68(6), 1703–1723. 10.1227/NEU.0b013e3182124690 21346655

[brb33107-bib-0022] De Benedictis, A. , Duffau, H. , Paradiso, B. , Grandi, E. , Balbi, S. , Granieri, E. , Colarusso, E. , Chioffi, F. , Marras, C. E. , & Sarubbo, S. (2014). Anatomo‐functional study of the temporo‐parieto‐occipital region: Dissection, tractographic and brain mapping evidence from a neurosurgical perspective. Journal of Anatomy, 225(2), 132–151. 10.1111/joa.12204 24975421 PMC4111924

[brb33107-bib-0023] De Benedictis, A. , Marras, C. E. , Petit, L. , & Sarubbo, S. (2022). The inferior fronto‐occipital fascicle: A century of controversies from anatomy theaters to operative neurosurgery. Journal of Neurosurgical Sciences, 65(6), 605–615. 10.23736/S0390-5616.21.05360-1 33940782

[brb33107-bib-0024] De Benedictis, A. , Sarubbo, S. , & Duffau, H. (2012). Subcortical surgical anatomy of the lateral frontal region: Human white matter dissection and correlations with functional insights provided by intraoperative direct brain stimulation: Laboratory investigation. Journal of Neurosurgery, 117(6), 1053–1069. 10.3171/2012.7.JNS12628 22998058

[brb33107-bib-0025] Dejerine, J. , & Dejerine‐Klumpke, A. (1895). Anatomie des centres nerveux. Tome 1. Rueff et Cie.

[brb33107-bib-0026] Dejerine, J. , & Dejerine‐Klumpke, A. (1901). Anatomie des centres nerveux. Tome 2. Rueff et Cie.

[brb33107-bib-0027] Duffau, H. (2009). Does post‐lesional subcortical plasticity exist in the human brain? Neuroscience Research, 65(2), 131–135. 10.1016/j.neures.2009.07.002 19616045

[brb33107-bib-0028] Duffau, H. (2014). The huge plastic potential of adult brain and the role of connectomics: New insights provided by serial mappings in glioma surgery. Cortex; A Journal Devoted to the Study of the Nervous System and Behavior, 58, 325–337. 10.1016/j.cortex.2013.08.005 24050218

[brb33107-bib-0029] Duffau, H. (2018). The error of Broca: From the traditional localizationist concept to a connectomal anatomy of human brain. Journal of Chemical Neuroanatomy, 89, 73–81. 10.1016/j.jchemneu.2017.04.003 28416459

[brb33107-bib-0030] Duffau, H. , de Schotten, M. T. , & Mandonnet, E. (2008). White matter functional connectivity as an additional landmark for dominant temporal lobectomy. Journal of Neurology, Neurosurgery & Psychiatry, 79(5), 492–495. 10.1136/jnnp.2007.121004 18408087

[brb33107-bib-0031] Duffau, H. , Lopes, M. , Arthuis, F. , Bitar, A. , & Sichez, J.‐P. (2005). Contribution of intraoperative electrical stimulations in surgery of low grade gliomas: A comparative study between two series without (1985–96) and with (1996–2003) functional mapping in the same institution. Journal of Neurology, Neurosurgery, and Psychiatry, 76, 7. 10.1136/jnnp.2004.048520 PMC1739650 15897509

[brb33107-bib-0032] Dziedzic, T. A. , Bala, A. , & Marchel, A. (2022). Anatomical aspects of the insula, opercula and peri‐insular white matter for a transcortical approach to insular glioma resection. Neurosurgical Review, 45(1), 793–806. 10.1007/s10143-021-01602-5 34292438 PMC8827298

[brb33107-bib-0033] Ellmore, T. M. , Beauchamp, M. S. , O'Neill, T. J. , Dreyer, S. , & Tandon, N. (2009). Relationships between essential cortical language sites and subcortical pathways. Journal of Neurosurgery, 111(4), 755–766. 10.3171/2009.3.JNS081427 19374498

[brb33107-bib-0034] Fang, S. , Liang, Y. , Li, L. , Wang, L. , Fan, X. , Wang, Y. , & Jiang, T. (2021). Tumor location‐based classification of surgery‐related language impairments in patients with glioma. Journal of Neuro‐Oncology, 155(2), 143–152. 10.1007/s11060-021-03858-9 34599481

[brb33107-bib-0035] Fernández‐Miranda, J. C. , Wang, Y. , Pathak, S. , Stefaneau, L. , Verstynen, T. , & Yeh, F.‐C. (2015). Asymmetry, connectivity, and segmentation of the arcuate fascicle in the human brain. Brain Structure and Function, 220(3), 1665–1680. 10.1007/s00429-014-0751-7 24633827

[brb33107-bib-0036] Forkel, S. J. , Friedrich, P. , Thiebaut de Schotten, M. , & Howells, H. (2021). White matter variability, cognition, and disorders: A systematic review. Brain Structure and Function, 227, 529–544. 10.1007/s00429-021-02382-w 34731328 PMC8844174

[brb33107-bib-0037] Forkel, S. J. , Rogalski, E. , Drossinos Sancho, N. , D'Anna, L. , Luque Laguna, P. , Sridhar, J. , Dell'Acqua, F. , Weintraub, S. , Thompson, C. , Mesulam, M.‐M. , & Catani, M. (2020). Anatomical evidence of an indirect pathway for word repetition. Neurology, 94(6), e594–e606. 10.1212/WNL.0000000000008746 31996450 PMC7136066

[brb33107-bib-0038] Forkel, S. J. , Thiebaut de Schotten, M. , Dell'Acqua, F. , Kalra, L. , Murphy, D. G. M. , Williams, S. C. R. , & Catani, M. (2014). Anatomical predictors of aphasia recovery: A tractography study of bilateral perisylvian language networks. Brain, 137(7), 2027–2039. 10.1093/brain/awu113 24951631

[brb33107-bib-0039] Frey, S. , Campbell, J. S. W. , Pike, G. B. , & Petrides, M. (2008). Dissociating the human language pathways with high angular resolution diffusion fiber tractography. Journal of Neuroscience, 28(45), 11435–11444. 10.1523/JNEUROSCI.2388-08.2008 18987180 PMC6671318

[brb33107-bib-0040] Fridriksson, J. , Kjartansson, O. , Morgan, P. S. , Hjaltason, H. , Magnusdottir, S. , Bonilha, L. , & Rorden, C. (2010). Impaired speech repetition and left parietal lobe damage. Journal of Neuroscience, 30(33), 11057–11061. 10.1523/JNEUROSCI.1120-10.2010 20720112 PMC2936270

[brb33107-bib-0041] Friederici, A. D. (2009). Pathways to language: Fiber tracts in the human brain. Trends in Cognitive Sciences, 13(4), 175–181. 10.1016/j.tics.2009.01.001 19223226

[brb33107-bib-0042] Frost, J. A. (1999). Language processing is strongly left lateralized in both sexes: Evidence from functional MRI. Brain, 122(2), 199–208. 10.1093/brain/122.2.199 10071049

[brb33107-bib-0043] Geschwind, N. (1965). Disconnexion syndromes in animals and man. II. Brain, 88(3), 585–644. 5318824 10.1093/brain/88.3.585

[brb33107-bib-0044] Geschwind, N. (1970). The organization of language and the brain. 10.1126/science.170.3961.940 5475022

[brb33107-bib-0045] Geva, S. , Correia, M. M. , & Warburton, E. A. (2015). Contributions of bilateral white matter to chronic aphasia symptoms as assessed by diffusion tensor MRI. Brain and Language, 150, 117–128. 10.1016/j.bandl.2015.09.001 26401977 PMC4669306

[brb33107-bib-0046] Gharabaghi, A. , Kunath, F. , Erb, M. , Saur, R. , Heckl, S. , Tatagiba, M. , Grodd, W. , & Karnath, H.‐O. (2009). Perisylvian white matter connectivity in the human right hemisphere. BMC Neuroscience, 10(1), 15. 10.1186/1471-2202-10-15 19257886 PMC2653039

[brb33107-bib-0047] Glasser, M. F. , & Rilling, J. K. (2008). DTI tractography of the human brain's language pathways. Cerebral Cortex, 18(11), 2471–2482. 10.1093/cercor/bhn011 18281301

[brb33107-bib-0048] Griffiths, J. D. , Marslen‐Wilson, W. D. , Stamatakis, E. A. , & Tyler, L. K. (2013). Functional organization of the neural language system: Dorsal and ventral pathways are critical for syntax. Cerebral Cortex, 23(1), 139–147. 10.1093/cercor/bhr386 22275482 PMC3601415

[brb33107-bib-0049] Grosse Wiesmann, C. , Schreiber, J. , Singer, T. , Steinbeis, N. , & Friederici, A. D. (2017). White matter maturation is associated with the emergence of Theory of Mind in early childhood. Nature Communications, 8(1), 14692. 10.1038/ncomms14692 PMC5364393 28322222

[brb33107-bib-0050] Güngör, A. , Baydin, S. , Middlebrooks, E. H. , Tanriover, N. , Isler, C. , & Rhoton, A. L. (2017). The white matter tracts of the cerebrum in ventricular surgery and hydrocephalus. Journal of Neurosurgery, 126(3), 945–971. 10.3171/2016.1.JNS152082 27257832

[brb33107-bib-0051] He, B. J. , Snyder, A. Z. , Vincent, J. L. , Epstein, A. , Shulman, G. L. , & Corbetta, M. (2007). Breakdown of functional connectivity in frontoparietal networks underlies behavioral deficits in spatial neglect. Neuron, 53(6), 905–918. 10.1016/j.neuron.2007.02.013 17359924

[brb33107-bib-0052] Herbet, G. , Lafargue, G. , Bonnetblanc, F. , Moritz‐Gasser, S. , de Champfleur, N. M. , & Duffau, H. (2014). Inferring a dual‐stream model of mentalizing from associative white matter fibres disconnection. Brain, 16, 944–959. 10.1093/brain/awt370 24519980

[brb33107-bib-0053] Herbet, G. , Maheu, M. , Costi, E. , Lafargue, G. , & Duffau, H. (2016). Mapping neuroplastic potential in brain‐damaged patients. Brain, 139(3), 829–844. 10.1093/brain/awv394 26912646

[brb33107-bib-0054] Hervey‐Jumper, S. L. , & Berger, M. S. (2016). Maximizing safe resection of low‐ and high‐grade glioma. Journal of Neuro‐Oncology, 130(2), 269–282. 10.1007/s11060-016-2110-4 27174197

[brb33107-bib-0055] Holland, S. K. , Vannest, J. , Mecoli, M. , Jacola, L. M. , Tillema, J.‐M. , Karunanayaka, P. R. , Schmithorst, V. J. , Yuan, W. , Plante, E. , & Byars, A. W. (2007). Functional MRI of language lateralization during development in children. International Journal of Audiology, 46(9), 533–551. 10.1080/14992020701448994 17828669 PMC2763431

[brb33107-bib-0056] Hope, T. M. H. , Seghier, M. L. , Prejawa, S. , Leff, A. P. , & Price, C. J. (2016). Distinguishing the effect of lesion load from tract disconnection in the arcuate and uncinate fasciculi. Neuroimage, 125, 1169–1173. 10.1016/j.neuroimage.2015.09.025 26388553 PMC4692449

[brb33107-bib-0057] Ivanova, M. V. , Isaev, D. Y. U. , Dragoy, O. V. , Akinina, Y. S. , Petrushevskiy, A. G. , Fedina, O. N. , Shklovsky, V. M. , & Dronkers, N. F. (2016). Diffusion‐tensor imaging of major white matter tracts and their role in language processing in aphasia. Cortex; A Journal Devoted to the Study of the Nervous System and Behavior, 85, 165–181. 10.1016/j.cortex.2016.04.019 27289586

[brb33107-bib-0058] Janssen, N. , Kessels, R. P. C. , Mars, R. B. , Llera, A. , Beckmann, C. F. , & Roelofs, A. (2023). Dissociating the functional roles of arcuate fasciculus subtracts in speech production. Cerebral Cortex, 33(6), 2539–2547. 10.1093/cercor/bhac224 35709759 PMC10016035

[brb33107-bib-0059] Jiang, J. , Zhao, Y.‐J. , Hu, X.‐Y. , Du, M.‐Y. , Chen, Z.‐Q. , Wu, M. , Li, K.‐M. , Zhu, H.‐Y. , Kumar, P. , & Gong, Q.‐Y. (2017). Microstructural brain abnormalities in medication‐free patients with major depressive disorder: A systematic review and meta‐analysis of diffusion tensor imaging. Journal of Psychiatry & Neuroscience, 42(3), 150–163. 10.1503/jpn.150341 27780031 PMC5403660

[brb33107-bib-0060] Klingler, J. (1935). Erleichterung des makroskopischen Praeparation des Gehirns durch den Gefrierprozess. Schweizer Archiv Für Neurologie Und Psychiatrie, 36, 247–256.

[brb33107-bib-0061] Kumar, D. R. , Aslinia, F. , Yale, S. H. , & Mazza, J. J. (2011). Jean‐Martin Charcot: The father of neurology. Clinical Medicine & Research, 9, 49–46. 10.3121/cmr.2009.883 PMC3064755 20739583

[brb33107-bib-0062] Kümmerer, D. , Hartwigsen, G. , Kellmeyer, P. , Glauche, V. , Mader, I. , Klöppel, S. , Suchan, J. , Karnath, H.‐O. , Weiller, C. , & Saur, D. (2013). Damage to ventral and dorsal language pathways in acute aphasia. Brain, 136(2), 619–629. 10.1093/brain/aws354 23378217 PMC3572927

[brb33107-bib-0063] Lawes, I. N. C. , Barrick, T. R. , Murugam, V. , Spierings, N. , Evans, D. R. , Song, M. , & Clark, C. A. (2008). Atlas‐based segmentation of white matter tracts of the human brain using diffusion tensor tractography and comparison with classical dissection. Neuroimage, 39(1), 62–79. 10.1016/j.neuroimage.2007.06.041 17919935

[brb33107-bib-0064] Leclercq, D. , Duffau, H. , Delmaire, C. , Capelle, L. , Gatignol, P. , Ducros, M. , Chiras, J. , & Lehéricy, S. (2010). Comparison of diffusion tensor imaging tractography of language tracts and intraoperative subcortical stimulations. Journal of Neurosurgery, 112(3), 503–511. 10.3171/2009.8.JNS09558 19747052

[brb33107-bib-0065] Leemans, A. (2019). Diffusion MRI of the brain: The naked truth. NMR in Biomedicine, 32(4), e4084. 10.1002/nbm.4084 30791163

[brb33107-bib-0066] López‐Barroso, D. , Catani, M. , Ripollés, P. , Dell'Acqua, F. , Rodríguez‐Fornells, A. , & de Diego‐Balaguer, R. (2013). Word learning is mediated by the left arcuate fasciculus. Proceedings of the National Academy of Sciences, 110(32), 13168–13173. 10.1073/pnas.1301696110 PMC3740909 23884655

[brb33107-bib-0067] Loui, P. , Alsop, D. , & Schlaug, G. (2009). Tone deafness: A new disconnection syndrome? Journal of Neuroscience, 29(33), 10215–10220. 10.1523/JNEUROSCI.1701-09.2009 19692596 PMC2747525

[brb33107-bib-0068] Loui, P. , Li, H. C. , & Schlaug, G. (2011). White matter integrity in right hemisphere predicts pitch‐related grammar learning. Neuroimage, 55(2), 500–507. 10.1016/j.neuroimage.2010.12.022 21168517 PMC3035724

[brb33107-bib-0069] Maldonado, I. L. , Moritz‐Gasser, S. , de Champfleur, N. M. , Bertram, L. , Moulinié, G. , & Duffau, H. (2011). Surgery for gliomas involving the left inferior parietal lobule: New insights into the functional anatomy provided by stimulation mapping in awake patients. Journal of Neurosurgery, 115(4), 770–779. 10.3171/2011.5.JNS112 21699481

[brb33107-bib-0070] Maldonado, I. L. , Moritz‐Gasser, S. , & Duffau, H. (2011). Does the left superior longitudinal fascicle subserve language semantics? A brain electrostimulation study. Brain Structure and Function, 216(3), 263–274. 10.1007/s00429-011-0309-x 21538022

[brb33107-bib-0071] Malik‐Moraleda, S. , Ayyash, D. , Gallée, J. , Affourtit, J. , Hoffmann, M. , Mineroff, Z. , Jouravlev, O. , & Fedorenko, E. (2022). An investigation across 45 languages and 12 language families reveals a universal language network. Nature Neuroscience, 25(8), 1014–1019. 10.1038/s41593-022-01114-5 35856094 PMC10414179

[brb33107-bib-0072] Mandonnet, E. , Martino, J. , Sarubbo, S. , Corrivetti, F. , Bouazza, S. , Bresson, D. , Duffau, H. , & Froelich, S. (2017). Neuronavigated fiber dissection with pial preservation: Laboratory model to simulate opercular approaches to insular tumors. World Neurosurgery, 98, 239–242. 10.1016/j.wneu.2016.10.020 27765721

[brb33107-bib-0073] Mandonnet, E. , Sarubbo, S. , & Petit, L. (2018). The nomenclature of human white matter association pathways: Proposal for a systematic taxonomic anatomical classification. Frontiers in Neuroanatomy, 12, 94. 10.3389/fnana.2018.00094 30459566 PMC6232419

[brb33107-bib-0074] Marchina, S. , Zhu, L. L. , Norton, A. , Zipse, L. , Wan, C. Y. , & Schlaug, G. (2011). Impairment of speech production predicted by lesion load of the left arcuate fasciculus. Stroke; A Journal of Cerebral Circulation, 42(8), 2251–2256. 10.1161/STROKEAHA.110.606103 PMC3167233 21719773

[brb33107-bib-0075] Martino, J. , De Witt Hamer, P. C. , Vergani, F. , Brogna, C. , de Lucas, E. M. , Vázquez‐Barquero, A. , García‐Porrero, J. A. , & Duffau, H. (2011). Cortex‐sparing fiber dissection: An improved method for the study of white matter anatomy in the human brain: Cortex‐sparing fiber dissection. Journal of Anatomy, 219(4), 531–541. 10.1111/j.1469-7580.2011.01414.x 21767263 PMC3196758

[brb33107-bib-0076] Martino, J. , Gomez, E. , Bilbao, J. L. , Dueñas, J. C. , & Vázquez‐Barquero, A. (2013). Cost‐utility of maximal safe resection of WHO grade II gliomas within eloquent areas. Acta Neurochirurgica, 155(1), 41–50. 10.1007/s00701-012-1541-8 23132374

[brb33107-bib-0077] Martino, J. , da Silva‐Freitas, R. , Caballero, H. , de Marco Lucas, E. , García‐Porrero, J. A. , & Vázquez‐Barquero, A. (2013). Fiber dissection and diffusion tensor imaging tractography study of the temporoparietal fiber intersection area. Operative Neurosurgery, 72(1), ons87–ons98. 10.1227/NEU.0b013e318274294b 23417154

[brb33107-bib-0078] Mayo, H. (1823). Anatomical and physiological commentaries, Number II. Underwood.

[brb33107-bib-0079] McDonald, C. R. , Ahmadi, M. E. , Hagler, D. J. , Tecoma, E. S. , Iragui, V. J. , Gharapetian, L. , Dale, A. M. , & Halgren, E. (2008). Diffusion tensor imaging correlates of memory and language impairments in temporal lobe epilepsy. Neurology, 71(23), 1869–1876. 10.1212/01.wnl.0000327824.05348.3b 18946001 PMC2676974

[brb33107-bib-0080] Meyer, L. , Cunitz, K. , Obleser, J. , & Friederici, A. D. (2014). Sentence processing and verbal working memory in a white‐matter‐disconnection patient. Neuropsychologia, 61, 190–196. 10.1016/j.neuropsychologia.2014.06.014 24953959

[brb33107-bib-0081] Moritz‐Gasser, S. , & Duffau, H. (2013). The anatomo‐functional connectivity of word repetition: Insights provided by awake brain tumor surgery. Frontiers in Human Neuroscience, 7, 00–00. 10.3389/fnhum.2013.00405 PMC3725408 23908617

[brb33107-bib-0082] Mullen, K. M. , Vohr, B. R. , Katz, K. H. , Schneider, K. C. , Lacadie, C. , Hampson, M. , Makuch, R. W. , Reiss, A. L. , Constable, R. T. , & Ment, L. R. (2011). Preterm birth results in alterations in neural connectivity at age 16 years. Neuroimage, 54(4), 2563–2570. 10.1016/j.neuroimage.2010.11.019 21073965 PMC3020252

[brb33107-bib-0083] Nakajima, R. , Kinoshita, M. , Shinohara, H. , & Nakada, M. (2019). The superior longitudinal fascicle: Reconsidering the fronto‐parietal neural network based on anatomy and function. Brain Imaging and Behavior, 14, 2817–2830. 10.1007/s11682-019-00187-4 31468374

[brb33107-bib-0084] Nakajima, R. , Yordanova, Y. N. , Duffau, H. , & Herbet, G. (2018). Neuropsychological evidence for the crucial role of the right arcuate fasciculus in the face‐based mentalizing network: A disconnection analysis. Neuropsychologia, 115, 179–187. 10.1016/j.neuropsychologia.2018.01.024 29360518

[brb33107-bib-0085] Papagno, C. , Comi, A. , Riva, M. , Bizzi, A. , Vernice, M. , Casarotti, A. , Fava, E. , & Bello, L. (2017). Mapping the brain network of the phonological loop: The phonological loop brain network. Human Brain Mapping, 38(6), 3011–3024. 10.1002/hbm.23569 28321956 PMC6866778

[brb33107-bib-0086] Papoutsi, M. , Stamatakis, E. A. , Griffiths, J. , Marslen‐Wilson, W. D. , & Tyler, L. K. (2011). Is left fronto‐temporal connectivity essential for syntax? Effective connectivity, tractography and performance in left‐hemisphere damaged patients. Neuroimage, 58(2), 656–664. 10.1016/j.neuroimage.2011.06.036 21722742

[brb33107-bib-0087] Peraud, A. , Ilmberger, J. , & Reulen, H.‐J. (2004). Surgical resection of gliomas WHO grade II and III located in the opercular region. Acta Neurochirurgica, 146(1), 9–18. 10.1007/s00701-003-0165-4 14740260

[brb33107-bib-0088] Phillips, O. R. , Clark, K. A. , Woods, R. P. , Subotnik, K. L. , Asarnow, R. F. , Nuechterlein, K. H. , Toga, A. W. , & Narr, K. L. (2011). Topographical relationships between arcuate fasciculus connectivity and cortical thickness. Human Brain Mapping, 32(11), 1788–1801. 10.1002/hbm.21147 20886580 PMC3071430

[brb33107-bib-0089] Plaza, M. , Gatignol, P. , Leroy, M. , & Duffau, H. (2009). Speaking without Broca's area after tumor resection. Neurocase, 15(4), 294–310. 10.1080/13554790902729473 19274574

[brb33107-bib-0090] Porto de Oliveira, J. V. M. , Raquelo‐Menegassio, A. F. , & Maldonado, I. L. (2021). What's your name again? A review of the superior longitudinal and arcuate fasciculus evolving nomenclature. Clinical Anatomy, 34(7), 1101–1110. 10.1002/ca.23764 34218465

[brb33107-bib-0091] Psomiades, M. , Fonteneau, C. , Mondino, M. , Luck, D. , Haesebaert, F. , Suaud‐Chagny, M.‐F. , & Brunelin, J. (2016). Integrity of the arcuate fasciculus in patients with schizophrenia with auditory verbal hallucinations: A DTI‐tractography study. NeuroImage: Clinical, 12, 970–975. 10.1016/j.nicl.2016.04.013 27995063 PMC5153606

[brb33107-bib-0092] Reijmer, Y. D. , Leemans, A. , Heringa, S. M. , Wielaard, I. , Jeurissen, B. , Koek, H. L. , & Biessels, G. J. (2012). Improved sensitivity to cerebral white matter abnormalities in Alzheimer's disease with spherical deconvolution based tractography. PLoS One, 7(8), e44074. 10.1371/journal.pone.0044074 22952880 PMC3432077

[brb33107-bib-0093] Reil, J. C. (1809). Die Sylvische Grube oder das Thal, das gestreifte grobe hirnganglium, dessen kapsel und die seitentheile des grobn gehirns. Archiv Für Die Physiologie, 9, 195–208.

[brb33107-bib-0094] Reil, J. C. (1812). Die vördere commissur im groben gehirn. Archiv Für Die Physiologie, 11, 89–100.

[brb33107-bib-0095] Rojkova, K. , Volle, E. , Urbanski, M. , Humbert, F. , Dell'Acqua, F. , & Thiebaut de Schotten, M. (2016). Atlasing the frontal lobe connections and their variability due to age and education: A spherical deconvolution tractography study. Brain Structure and Function, 221(3), 1751–1766. 10.1007/s00429-015-1001-3 25682261

[brb33107-bib-0096] Rolland, A. , Herbet, G. , & Duffau, H. (2018). Awake surgery for gliomas within the right inferior parietal lobule: New insights into the functional connectivity gained from stimulation mapping and surgical implications. World Neurosurgery, 112, e393–e406. 10.1016/j.wneu.2018.01.053 29355798

[brb33107-bib-0097] Roux, F.‐E. , Dufor, O. , Lauwers‐Cances, V. , Boukhatem, L. , Brauge, D. , Draper, L. , Lotterie, J.‐A. , & Démonet, J.‐F. (2011). Electrostimulation mapping of spatial neglect. Neurosurgery, 69(6), 1218–1231. 10.1227/NEU.0b013e31822aefd2 22067336

[brb33107-bib-0098] Sanai, N. , Polley, M.‐Y. , McDermott, M. W. , Parsa, A. T. , & Berger, M. S. (2011). An extent of resection threshold for newly diagnosed glioblastomas. Journal of Neurosurgery, 115(1), 3–8. 10.3171/2011.2.JNS10998 21417701

[brb33107-bib-0099] Sarubbo, S. , De Benedictis, A. , Merler, S. , Mandonnet, E. , Balbi, S. , Granieri, E. , & Duffau, H. (2015). Towards a functional atlas of human white matter: Functional atlas of white matter. Human Brain Mapping, 36(8), 3117–3136. 10.1002/hbm.22832 25959791 PMC6869563

[brb33107-bib-0100] Sarubbo, S. , De Benedictis, A. , Merler, S. , Mandonnet, E. , Barbareschi, M. , Dallabona, M. , Chioffi, F. , & Duffau, H. (2016). Structural and functional integration between dorsal and ventral language streams as revealed by blunt dissection and direct electrical stimulation: Anatomo‐functional integration of language. Human Brain Mapping, 37(11), 3858–3872. 10.1002/hbm.23281 27258125 PMC6867442

[brb33107-bib-0101] Sarubbo, S. , Tate, M. , De Benedictis, A. , Merler, S. , Moritz‐Gasser, S. , Herbet, G. , & Duffau, H. (2020). Mapping critical cortical hubs and white matter pathways by direct electrical stimulation: An original functional atlas of the human brain. Neuroimage, 205, 116237. 10.1016/j.neuroimage.2019.116237 31626897 PMC7217287

[brb33107-bib-0102] Saur, D. , Kreher, B. W. , Schnell, S. , Kümmerer, D. , Kellmeyer, P. , Vry, M.‐S. , Umarova, R. , Musso, M. , Glauche, V. , Abel, S. , Huber, W. , Rijntjes, M. , Hennig, J. , & Weiller, C. (2008). Ventral and dorsal pathways for language. Proceedings of the National Academy of Sciences, 105(46), 18035–18040. 10.1073/pnas.0805234105 PMC2584675 19004769

[brb33107-bib-0103] Saygin, Z. M. , Norton, E. S. , Osher, D. E. , Beach, S. D. , Cyr, A. B. , Ozernov‐Palchik, O. , Yendiki, A. , Fischl, B. , Gaab, N. , & Gabrieli, J. D. E. (2013). Tracking the roots of reading ability: White matter volume and integrity correlate with phonological awareness in prereading and early‐reading kindergarten children. Journal of Neuroscience, 33(33), 13251–13258. 10.1523/JNEUROSCI.4383-12.2013 23946384 PMC3742917

[brb33107-bib-0104] Sierpowska, J. , Gabarrós, A. , Fernandez‐Coello, A. , Camins, À. , Castañer, S. , Juncadella, M. , Morís, J. , & Rodríguez‐Fornells, A. (2017). Words are not enough: Nonword repetition as an indicator of arcuate fasciculus integrity during brain tumor resection. Journal of Neurosurgery, 126(2), 435–445. 10.3171/2016.2.JNS151592 27177174

[brb33107-bib-0105] Swanson, L. W. (2015). Neuroanatomical terminology: A lexicon of classical origins and historical foundations. Oxford University Press.

[brb33107-bib-0106] Szaflarski, J. P. , Binder, J. R. , Possing, E. T. , McKiernan, K. A. , Ward, B. D. , & Hammeke, T. A. (2002). Language lateralization in left‐handed and ambidextrous people: FMRI data. Neurology, 59(2), 238–244. 10.1212/WNL.59.2.238 12136064

[brb33107-bib-0107] Teubner‐Rhodes, S. , Vaden, K. I. , Cute, S. L. , Yeatman, J. D. , Dougherty, R. F. , & Eckert, M. A. (2016). Aging‐resilient associations between the arcuate fasciculus and vocabulary knowledge: Microstructure or morphology? Journal of Neuroscience, 36(27), 7210–7222. 10.1523/JNEUROSCI.4342-15.2016 27383595 PMC4938863

[brb33107-bib-0108] Thiebaut de Schotten, M. (2005). Direct evidence for a parietal‐frontal pathway subserving spatial awareness in humans. Science, 309(5744), 2226–2228. 10.1126/science.1116251 16195465

[brb33107-bib-0109] Thiebaut de Schotten, M. , Dell'Acqua, F. , Valabregue, R. , & Catani, M. (2012). Monkey to human comparative anatomy of the frontal lobe association tracts. Cortex; A Journal Devoted to the Study of the Nervous System and Behavior, 48(1), 82–96. 10.1016/j.cortex.2011.10.001 22088488

[brb33107-bib-0110] Umarova, R. M. , Saur, D. , Schnell, S. , Kaller, C. P. , Vry, M.‐S. , Glauche, V. , Rijntjes, M. , Hennig, J. , Kiselev, V. , & Weiller, C. (2010). Structural connectivity for visuospatial attention: Significance of ventral pathways. Cerebral Cortex, 20(1), 121–129. 10.1093/cercor/bhp086 19406904

[brb33107-bib-0111] Vandermosten, M. , Boets, B. , Poelmans, H. , Sunaert, S. , Wouters, J. , & Ghesquiere, P. (2012). A tractography study in dyslexia: Neuroanatomic correlates of orthographic, phonological and speech processing. Brain, 135(3), 935–948. 10.1093/brain/awr363 22327793

[brb33107-bib-0112] van Geemen, K. , Herbet, G. , Moritz‐Gasser, S. , & Duffau, H. (2014). Limited plastic potential of the left ventral premotor cortex in speech articulation: Evidence From intraoperative awake mapping in glioma patients: Ventral Premotor Cortex and Speech. Human Brain Mapping, 35(4), 1587–1596. 10.1002/hbm.22275 23616288 PMC6869841

[brb33107-bib-0113] Vaquero, L. , Ramos‐Escobar, N. , Cucurell, D. , François, C. , Putkinen, V. , Segura, E. , Huotilainen, M. , Penhune, V. , & Rodríguez‐Fornells, A. (2021). Arcuate fasciculus architecture is associated with individual differences in pre‐attentive detection of unpredicted music changes. Neuroimage, 229, 117759. 10.1016/j.neuroimage.2021.117759 33454403

[brb33107-bib-0114] Vassal, F. , Schneider, F. , Boutet, C. , Jean, B. , Sontheimer, A. , & Lemaire, J.‐J. (2016). Combined DTI tractography and functional MRI study of the language connectome in healthy volunteers: Extensive mapping of white matter fascicles and cortical activations. PLoS One, 11(3), e0152614. 10.1371/journal.pone.0152614 27029050 PMC4814138

[brb33107-bib-0115] Vassal, F. , Schneider, F. , Sontheimer, A. , Lemaire, J.‐J. , & Nuti, C. (2013). Intraoperative visualisation of language fascicles by diffusion tensor imaging‐based tractography in glioma surgery. Acta Neurochirurgica, 155(3), 437–448. 10.1007/s00701-012-1580-1 23254890

[brb33107-bib-0116] Vavassori, L. , Sarubbo, S. , & Petit, L. (2021). Hodology of the superior longitudinal system of the human brain: A historical perspective, the current controversies, and a proposal. Brain Structure and Function, 226, 1363–1384. 33881634 10.1007/s00429-021-02265-0

[brb33107-bib-0117] Vigneau, M. , Beaucousin, V. , Hervé, P. Y. , Duffau, H. , Crivello, F. , Houdé, O. , Mazoyer, B. , & Tzourio‐Mazoyer, N. (2006). Meta‐analyzing left hemisphere language areas: Phonology, semantics, and sentence processing. Neuroimage, 30(4), 1414–1432. 10.1016/j.neuroimage.2005.11.002 16413796

[brb33107-bib-0118] Monakow, C. (1897). Gehirnpathologie: Allgemeine Einleitung, Localisation, Gehirnblutungen, Verstopfung der Hirnarterien.

[brb33107-bib-0119] Wang, J. , Marchina, S. , Norton, A. C. , Wan, C. Y. , & Schlaug, G. (2013). Predicting speech fluency and naming abilities in aphasic patients. Frontiers in Human Neuroscience, 7, 831. 10.3389/fnhum.2013.00831 24339811 PMC3857577

[brb33107-bib-0120] Wang, S. , Van der Haegen, L. , Tao, L. , & Cai, Q. (2019). Brain functional organization associated with language lateralization. Cerebral Cortex, 29(10), 4312–4320. 10.1093/cercor/bhy313 30561523

[brb33107-bib-0121] Wernicke, C. (1874). Der aphasische Symptomencomplex: Eine psychologische Studie auf anatomischer Basis. Breslau, Cohn & Weigert.

[brb33107-bib-0122] Wilson, S. M. , Galantucci, S. , Tartaglia, M. C. , Rising, K. , Patterson, D. K. , Henry, M. L. , Ogar, J. M. , DeLeon, J. , Miller, B. L. , & Gorno‐Tempini, M. L. (2011). Syntactic processing depends on dorsal language tracts. Neuron, 72(2), 397–403. 10.1016/j.neuron.2011.09.014 22017996 PMC3201770

[brb33107-bib-0123] Yagmurlu, K. , Middlebrooks, E. H. , Tanriover, N. , & Rhoton, A. L. (2016). Fiber tracts of the dorsal language stream in the human brain. Journal of Neurosurgery, 124(5), 1396–1405. 10.3171/2015.5.JNS15455 26587654

[brb33107-bib-0124] Yeatman, J. D. , Dougherty, R. F. , Ben‐Shachar, M. , & Wandell, B. A. (2012). Development of white matter and reading skills. Proceedings of the National Academy of Sciences, 109(44), E3045–E3053. 10.1073/pnas.1206792109 PMC3497768 23045658

[brb33107-bib-0125] Yeatman, J. D. , Dougherty, R. F. , Myall, N. J. , Wandell, B. A. , & Feldman, H. M. (2012). Tract profiles of white matter properties: Automating fiber‐tract quantification. PLoS One, 7(11), e49790. 10.1371/journal.pone.0049790 23166771 PMC3498174

[brb33107-bib-0126] Yeatman, J. D. , Dougherty, R. F. , Rykhlevskaia, E. , Sherbondy, A. J. , Deutsch, G. K. , Wandell, B. A. , & Ben‐Shachar, M. (2011). Anatomical properties of the arcuate fasciculus predict phonological and reading skills in children. Journal of Cognitive Neuroscience, 23(11), 3304–3317. 10.1162/jocn_a_00061 21568636 PMC3214008

[brb33107-bib-0127] Yordanova, Y. N. , Duffau, H. , & Herbet, G. (2017). Neural pathways subserving face‐based mentalizing. Brain Structure and Function, 222(7), 3087–3105. 10.1007/s00429-017-1388-0 28243761

[brb33107-bib-0128] Zarino, B. , Di Cristofori, A. , Fornara, G. A. , Bertani, G. A. , Locatelli, M. , Caroli, M. , Rampini, P. , Cogiamanian, F. , Crepaldi, D. , & Carrabba, G. (2020). Long‐term follow‐up of neuropsychological functions in patients with high grade gliomas: Can cognitive status predict patient's outcome after surgery? Acta Neurochirurgica, 162(4), 803–812. 10.1007/s00701-020-04230-y 31993749

[brb33107-bib-0129] Zemmoura, I. , Serres, B. , Andersson, F. , Barantin, L. , Tauber, C. , Filipiak, I. , Cottier, J.‐P. , Venturini, G. , & Destrieux, C. (2014). FIBRASCAN: A novel method for 3D white matter tract reconstruction in MR space from cadaveric dissection. Neuroimage, 103, 106–118. 10.1016/j.neuroimage.2014.09.016 25234114

[brb33107-bib-0130] Zigiotto, L. , Annicchiarico, L. , Corsini, F. , Vitali, L. , Falchi, R. , Dalpiaz, C. , Rozzanigo, U. , Barbareschi, M. , Avesani, P. , Papagno, C. , Duffau, H. , Chioffi, F. , & Sarubbo, S. (2020). Effects of supra‐total resection in neurocognitive and oncological outcome of high‐grade gliomas comparing asleep and awake surgery. Journal of Neuro‐Oncology, 148, 97–108. 32303975 10.1007/s11060-020-03494-9

[brb33107-bib-0131] Zigiotto, L. , Vavassori, L. , Annicchiarico, L. , Corsini, F. , Avesani, P. , Rozzanigo, U. , Sarubbo, S. , & Papagno, C. (2022). Segregated circuits for phonemic and semantic fluency: A novel patient‐tailored disconnection study. NeuroImage: Clinical, 36, 103149. 10.1016/j.nicl.2022.103149 35970113 PMC9400120

